# Antibacterial, Anti-Inflammatory, and Wound-Healing Effects of *Micromeria fruticosa* Essential Oil-Loaded Nanoemulsion in MRSA-Infected Diabetic Wounds: In Vitro and In Vivo Evaluation

**DOI:** 10.3390/pharmaceutics18101252

**Published:** 2026-10-01

**Authors:** Azad Mammadov, Mehmet Çimentepe, Özge Öztürk Çimentepe, Serpil Demirci Kayiran, Umay Merve Güven Bolgen, Tilbe Çevikelli, Ahmet Alper Öztürk, Muhammet Bahaeddin Dörtbudak, Akın Yiğin, Metin Yildirim

**Affiliations:** 1 Department of Medical Pharmacology, Faculty of Medicine, Kafkas University, Kars 36100, Türkiye; azad.mammadov@kafkas.edu.tr; 2 Department of Pharmaceutical Microbiology, Faculty of Pharmacy, Harran University, Sanliurfa 63300, Türkiye; 3 Department of Pharmacology, Faculty of Pharmacy, Harran University, Sanliurfa 63300, Türkiye; 4 Department of Pharmaceutical Botany, Faculty of Pharmacy, Çukurova University, Adana 01330, Türkiye; 5 Department of Pharmaceutical Technology, Faculty of Pharmacy, Çukurova University, Adana 01330, Türkiye; 6 Department of Pharmaceutical Technology, Faculty of Pharmacy, Bahçeşehir University, Istanbul 34353, Türkiye; 7 Department of Pharmaceutical Technology, Faculty of Pharmacy, Anadolu University, Eskisehir 26470, Türkiye; 8 Department of Pathology, Faculty of Veterinary Medicine, Harran University, Sanliurfa 63000, Türkiye; 9 Department of Genetics, Faculty of Veterinary Medicine, Harran University, Sanliurfa 63200, Türkiye; 10 Department of Biochemistry, Faculty of Pharmacy, Çukurova University, Adana 01330, Türkiye

**Keywords:** *Micromeria fruticosa*, nanoemulsion, methicillin-resistant *Staphylococcus aureus*, diabetic foot ulcer, wound healing, essential oil, pulegone

## Abstract

**Background/Objectives:** Diabetic foot ulcers infected with methicillin-resistant *Staphylococcus aureus* (MRSA) heal poorly and respond inadequately to conventional antibiotic therapy. This study developed a *Micromeria fruticosa* essential oil-loaded nanoemulsion (Mf-NE) as a topical treatment for such wounds. **Methods:** The essential oil was obtained by hydrodistillation and analyzed by GC–MS. Nanoemulsions were developed from pseudoternary phase diagrams using oleic acid and a Cremophor^®^ EL/Tween 80–Transcutol^®^ system (2:1 surfactant/cosurfactant) and characterized physicochemically. Antibacterial activity against MRSA (ATCC 43300) was assessed by agar well diffusion and microdilution, antioxidant activity by DPPH and ABTS assays, and cytocompatibility by MTT assay on L929 fibroblasts. Efficacy was evaluated over 12 days in a streptozotocin-induced MRSA-infected diabetic wound model in rats. **Results:** Pulegone (42.81%) and piperitenone (29.73%) were the major constituents. Incorporation of 5% (*w*/*w*) essential oil raised the droplet size from 214.5 ± 14.4 to 292.9 ± 9.9 nm; both formulations had a dermatologically acceptable pH and Newtonian flow behavior. Mf-NE produced a 16.1 mm inhibition zone and a MIC of 1.25 mg/mL, whereas NE produced no inhibition zone and had a MIC of 5 mg/mL. Radical scavenging reached 61.5% by DPPH at 10 mg/mL and 71.5% by ABTS at 0.2 mg/mL, and cell viability remained above 87%. The NE and Mf-NE formulations also demonstrated good biocompatibility in L929 fibroblast cells. Topical Mf-NE reduced the remaining wound area to 4.8 ± 0.9% of the initial area by day 12, compared with 21.0 ± 1.9% for Fucidin, 26.1 ± 1.9% for NE and 57.2 ± 1.3% for the untreated group; bacterial colonization was almost completely suppressed, TNF-α and IL-1β expression was significantly decreased, and composite histopathological scores were lower than in the untreated group (*p* = 0.0007). **Conclusions:** Mf-NE is a promising topical candidate for MRSA-infected diabetic wounds. Formulation-specific dermal and systemic safety requires further evaluation.

## 1. Introduction

Diabetes mellitus (DM) is one of the fastest-growing chronic metabolic disorders worldwide, and it places a growing burden on health systems. The International Diabetes Federation estimated that 536.6 million adults (10.5% of the population aged 20–79 years) were living with diabetes in 2021, and this number is expected to reach 783.2 million by 2045 [1]. Diabetic foot ulcers (DFUs) are among the most disabling chronic complications of diabetes, with a reported lifetime risk of 19–34% [2]. The prognosis after ulceration is poor. Recurrence rates reach 50–65% within 3–5 years, about 20% of patients eventually undergo lower-extremity amputation, and the 5-year mortality after a DFU approaches 50% [2]. Foot ulceration commonly results from peripheral neuropathy, ischemia, and repetitive minor trauma, and the diabetic foot is highly prone to bacterial colonization and infection. Infection is a principal factor that leads to hospitalization, amputation, and death [2,3].

The impaired immune response, microvascular dysfunction, and chronic hyperglycemia of the diabetic foot provide a suitable environment for opportunistic and multidrug-resistant pathogens. *Staphylococcus aureus* is a principal cause of diabetic foot infections, and its multidrug-resistant form, methicillin-resistant *S. aureus* (MRSA), has become increasingly common in foot ulcers, where it is associated with slower healing and a higher risk of amputation [4]. This problem is aggravated by the wider crisis of antimicrobial resistance (AMR), which was associated with an estimated 4.95 million deaths worldwide in 2019. MRSA alone was the single pathogen-drug combination responsible for more than 100,000 deaths directly attributable to AMR in that year [5]. Because the misuse and overuse of conventional antibiotics continue to reduce their efficacy and accelerate the emergence of resistant strains, new therapeutic strategies that can control drug-resistant infection and at the same time support wound healing are urgently needed.

*Micromeria fruticosa* L. (*M. fruticosa*) Druce belongs to the Lamiaceae family and is commonly referred to as stonemint. It is a Mediterranean species that generally grows in dry, warm conditions, particularly in stony and clay-based soils [6]. The leaves of the plant contain essential oil with a dominant spicy and aromatic odor. For this reason, the oil has been valued in perfumery and aromatherapy [7]. Essential oils are secondary metabolites, and their properties are not stable in every growing condition. Their yield, quality, and chemical composition may change according to climate, soil composition, geographical region, and soil moisture. In addition, the age of the plant, its developmental stage, the organ collected, and the time of harvest may affect the composition of the essential oil [8,9].

The essential oil of *M. fruticosa* includes different groups of terpenic compounds. Among them are monoterpene hydrocarbons such as β-pinene and D-limonene, oxygenated monoterpenes including pulegone and isomenthone, and sesquiterpene hydrocarbons such as bicyclogermacrene. Extracts obtained from *M. fruticosa* have also been reported to contain flavonoids, caffeic acid, and tannins. In addition to its phytochemical composition, several studies have described a range of biological activities for the essential oils of this species, including antimicrobial, anticancer, anti-inflammatory, antioxidant, antidepressant, anti-emetic, analgesic, antispasmodic, and cytotoxic effects [7,10,11,12,13,14,15,16].

In folk medicine, *M. fruticosa* has long been used for various complaints, including abdominal pain, diarrhea, colds and wounds [14]. *M. fruticosa* essential oil has been reported to inhibit clinically relevant bacteria, including MRSA, with minimum inhibitory concentrations of 3.1–6.3 mg/mL [13]. In addition, extracts of *M. fruticosa* accelerated wound healing and improved histological skin recovery in an experimental burn model, which indicates the antimicrobial and wound-healing potential of the plant for topical use [17]. Taken together, these properties address the two features that define an infected diabetic wound: the antibacterial activity reported against MRSA [13] is directed at the infectious component, while the accelerated closure and improved histological recovery reported for *M. fruticosa* in an experimental burn model [17] relate to the impaired repair process itself. This combination, together with the traditional use of the plant for wounds [14], was the basis for selecting *M. fruticosa* essential oil for the present study.

Despite these promising activities, the direct use of essential oils is limited by their high volatility, poor water solubility, and susceptibility to chemical degradation, all of which reduce their bioavailability and reproducibility [18]. Nanoemulsions, which are kinetically stable colloidal dispersions of nanometric oil droplets, have emerged as an effective delivery system to overcome these limitations. By encapsulating lipophilic compounds, nanoemulsions improve their physical and chemical stability, bioavailability, and controlled release, and they provide better antibacterial properties than conventional (coarse) emulsions [18,19]. In agreement with this, a clove oil nanoemulsion showed superior antibacterial and antibiofilm activity compared with the corresponding coarse emulsion [19]. Clove oil nanoemulsions also accelerated wound closure and improved histological repair in a burn wound model, and they have been proposed as promising candidates for chronic and diabetic wounds [20]. To the best of our knowledge, however, the formulation of *M. fruticosa* essential oil into a nanoemulsion and its evaluation against MRSA-infected diabetic wounds has not been reported.

In the present study, we aimed to develop *M. fruticosa* essential oil-loaded nanoemulsion formulations to control MRSA-associated infections that are commonly encountered in diabetic foot wounds and to promote the wound-healing process. For this purpose, the essential oil was obtained by hydrodistillation and its chemical composition was determined by GC–MS. A nanoemulsion system was then developed using pseudoternary phase diagrams and characterized in terms of droplet size, polydispersity index, zeta potential, conductivity, pH and rheological behavior. The in vitro antibacterial activity of the formulations against MRSA was evaluated by agar well diffusion and resazurin-based microdilution, and their free radical scavenging capacity was assessed by DPPH and ABTS assays. Finally, the therapeutic potential of the formulations was tested in an experimental MRSA-infected diabetic foot wound model, in which the wound area and the bacterial load in the wound tissue were monitored, and tissue repair was further evaluated by histopathological examination and by immunohistochemical staining and gene expression for TNF-α and IL-1β.

## 2. Materials and Methods

### 2.1. Plant Material

The aerial parts of *M. fruticosa* were collected from Kahramanmaraş, Türkiye. The plant material was identified by Dr. S. Demirci, and voucher specimens were deposited in the Herbarium of the Faculty of Pharmacy, Çukurova University, Adana, Türkiye, under the voucher number CUEF 2496.

### 2.2. Hydro-Distillation of M. fruticosa EOs

The aerial parts were dried in the shade at room temperature until they reached constant weight. For the isolation of essential oil, 50 g of the dried plant material was placed in a Clevenger apparatus (ISOLAB, Istanbul, Türkiye) with 500 mL of distilled water and hydrodistilled for 3 h. The extraction was carried out in three replicates. The oil yield was approximately 1.1 mL/100 g dry material. After hydrodistillation, the oil was separated, dried over anhydrous sodium sulfate, and stored at 4 °C. The sample was diluted with dichloromethane (1:3) before GC–MS analysis.

### 2.3. GC–MS Analysis of M. fruticosa EOs

We analyzed the essential oil samples of *M. fruticosa* by GC–MS. The system consisted of an Agilent 7890B gas chromatograph coupled to an Agilent 7010B mass spectrometer (Agilent Technologies Inc., Santa Clara, CA, USA). Electron impact ionization was set at 70 eV. Separation was made on a DB-WAX capillary column, 60 m × 0.25 mm i.d., with a film thickness of 0.25 μm (J&W Scientific, Folsom, CA, USA).

The injector and detector temperatures were 250 °C. Helium was used as the carrier gas at 1.4 mL/min. For each run, 1.0 μL of the essential oil sample was injected in split mode, with a split ratio of 20:1. The oven temperature was kept at 40 °C for 4 min, then raised to 250 °C at 5 °C/min and held there for 10 min. The mass spectrometer was used in full-scan mode, between *m*/*z* 40 and 400. The ion source temperature was 230 °C, and the quadrupole temperature was 150 °C.

The compounds were assigned by comparing their mass spectra with the NIST14 library. Retention indices were also calculated and checked against published RI values. The relative percentage of each compound was calculated from the normalized peak areas using NIST14 software.

### 2.4. Materials

Oleic acid, Cremophor^®^ EL, Tween 80 and other solvents were purchased from Sigma-Aldrich (Taufkirchen, Germany) and were of analytical grade. Transcutol^®^ P was obtained from Gattefossé (Saint-Priest, France). Ultrapure water was obtained using a Milli-Q purification system (Merck, Darmstadt, Germany) and used throughout the study.

### 2.5. Development of Essential Oil-Based Nanoemulsion Formulations

#### 2.5.1. Construction of Pseudoternary Phase Diagrams

Oleic acid was used as the oil phase in the preparation of nanoemulsions. Cremophor^®^ EL and Tween 80 were used as the surfactants (S), while a mixture of Transcutol^®^ P (highly purified pharmaceutical grade, ≥99.8% purity, Gattefossé, Saint-Priest, France) was employed as the cosurfactant (CoS). The Cremophor^®^ EL-Tween 80 mixture was prepared at a fixed ratio of 1:1 (*w*/*w*). Pseudoternary phase diagrams were constructed using different surfactant-to-cosurfactant (S_mix_) weight ratios of 1:1, 2:1, and 3:1. For each S_mix_ ratio, the proportion of oil phase to the S_mix_ was varied in range of 10:90, 20:80, 30:70, 40:60, 50:50, 60:40, 70:30, 80:20, 90:10 (*w*/*w*) respectively for development of 10 g nanoemulsion [21,22]. Pseudoternary phase diagrams were prepared through the water titration approach performed at room temperature under constant magnetic agitation. The resulting formulations were visually examined for transparency and uniformity. Phase diagram construction was carried out using specialized computer software, which enabled the identification and quantitative determination of the nanoemulsion regions [23].

#### 2.5.2. Preparation of Nanoemulsion Formulations

Nanoemulsion compositions were chosen from the area that has shown the largest nanoemulsion region in the pseudoternary phase diagram. Based on the optimized nanoemulsion system, formulations were prepared by the spontaneous emulsification method. *Micromeria* essential oil (5% (*w*/*w*)) was added in the oily phase of the optimized nanoemulsion system.

### 2.6. Characterization of Nanoemulsion Formulations

#### 2.6.1. Determination of Droplet Size and Size Distribution

Dynamic light scattering (DLS) was used to measure the droplet size and droplet size distribution of blank, *Micromeria* essential oil and containing nanoemulsions. The HORIBA SZ-100 DLS instrument (HORIBA, Kyoto, Japan) was used at scattering angles of 90 and 173°. Data were calculated as average diameter ± standard deviation (SD) of three consecutive analyses [24].

#### 2.6.2. Zeta Potential

Zeta potential measurements were performed using a HORIBA SZ-100 analyzer (HORIBA, Kyoto, Japan) equipped with simple folded capillary zeta cells. Prior to analysis, samples were allowed to equilibrate at the measurement temperature, and each determination was conducted under controlled conditions at 25 ± 1 °C. All measurements were carried out in triplicate, and the mean value was reported for subsequent evaluation [25].

#### 2.6.3. Determination of Conductivity

Electrical conductivity of the formulations was determined using the same HORIBA SZ-100 system fitted with simple folded capillary cells specifically designed for conductivity analysis. The measurements were taken three times at 25 ± 1 °C [26].

#### 2.6.4. pH Value

The pH values of the blank nanoemulsion and *Micromeria* essential oil-loaded nanoemulsion were determined using a calibrated digital pH meter (Mettler Toledo FiveGo, Greifensee, Switzerland). Measurements were performed by direct electrode immersion into the samples at 25 ± 1 °C. Data were represented as the mean ± SD (*n* = 3) [27].

#### 2.6.5. Viscosity and Rheological Measurement

The apparent viscosity of the blank nanoemulsion and *Micromeria* essential oil-loaded nanoemulsion were evaluated using a cone and plate rheometer (Brookfield DV3THACJ0, Brookfield Engineering Laboratories, Middleboro, MA, USA). Measurements were conducted under temperature-controlled conditions at 25 ± 1 °C, with shear rates corresponding to rotational speeds ranging from 10 to 100 rpm. All analyses were performed in triplicate (*n* = 3) [28].

### 2.7. Microbiological Assays

The antibacterial activity of the NE and Mf-NE formulations against methicillin-resistant *Staphylococcus aureus* (MRSA; ATCC 43300) was assessed using the agar well diffusion assay and the microdilution method [29,30]. Prior to the experiment, the MRSA culture was grown under appropriate conditions and a bacterial suspension equivalent to the 0.5 McFarland standard was prepared. The suspension was inoculated uniformly onto the surface of Mueller–Hinton agar (MHA) using a sterile swab. Wells 7 mm in diameter were cut into the agar under aseptic conditions, and 100 µL of the NE and Mf-NE systems was added to each well. A 30 µg cefoxitin disk was used as a positive control in order to confirm methicillin resistance phenotypically. After incubation, the resulting inhibition zones were measured, and antibacterial activity was evaluated by recording the zone diameters in millimeters (mm).

The antibacterial activities of the NE and Mf-NE systems were determined by the resazurin-based microdilution method. The MRSA strain was used as the test microorganism. Bacterial suspensions were adjusted to the 0.5 McFarland standard and combined in 96-well microplates with serial dilutions of the NE and Mf-NE ranging from 9.76 to 5000 µg/mL. The final bacterial density was adjusted to 1 × 10^5^ CFU/mL. Medium, bacteria and vancomycin controls were included in the experimental design. After incubation, 20 µL of sterile 0.01% (*w*/*v*) resazurin solution was added to each well and the plates were incubated for a further 3 h. The lowest nanoemulsion concentration at which resazurin retained its blue color was recorded as the MIC value.

### 2.8. Antioxidant Activity

The antioxidant activities of the nanoemulsion containing NE and Mf-NE were determined using the ABTS and DPPH free radical scavenging assays, based on a previously described method with some modifications [31]. For the DPPH assay, 0.5 mL of the NE and Mf-NE formulations at different concentrations was mixed with 2.5 mL of 0.1 mM DPPH solution. The mixture was incubated in the dark for 30 min, and at the end of the reaction the absorbance was measured spectrophotometrically at 517 nm.

For the ABTS assay, 7 mM ABTS solution and 2.45 mM potassium persulfate (K_2_S_2_O_8_) solution were combined in equal volumes, and the mixture was kept in the dark for 12 h to allow radical formation to be completed. A 0.5 mL aliquot of the formulations was mixed with 2.5 mL of the ABTS working solution, the mixture was allowed to react in the dark for 30 min, and the absorbance was determined at 734 nm. DPPH and ABTS radical scavenging activities were calculated as percentage inhibition (%).

### 2.9. Biocompatibility Test

The biocompatibility of NE and Mf-NE was assessed by means of an MTT-based cell viability assay using NCTC clone 929 (L929; CCL-1, ATCC) fibroblasts [32]. Briefly, L929 cells were distributed into 96-well culture plates at a concentration of 2 × 10^4^ cells/well and maintained under standard culture conditions until adequate cell adhesion was achieved. Each formulation was applied to L929 cells at final concentrations ranging from 0 to 50 μg/mL. Untreated cells (0 μg/mL) were used as the control group. Following treatment, the cells were maintained for 24 h at 37 °C in a humidified atmosphere containing 5% CO_2_. At the end of the incubation period, 0.5 mg/mL MTT solution was introduced into each well, and the plates were incubated for a further 3 h at 37 °C. During this period, viable metabolically active cells reduced MTT to insoluble formazan crystals. The formed crystals were then solubilized by adding 100 µL DMSO to each well. Absorbance values were measured at 570 nm using a microplate reader. Each experimental condition was analyzed in triplicate to ensure the reliability and reproducibility of the results.

### 2.10. In Vivo Experimental Studies

#### 2.10.1. Establishment of the MRSA-Infected Diabetic Foot Wound Model

All animal experiments were conducted in accordance with the National Research Council Guide for the Care and Use of Laboratory Animals. The study protocol was approved by the Local Ethics Committee for Animal Experiments of Harran University (HÜ-HADYEK-2026-00066). A total of 20 male Wistar albino rats (250–300 g) were obtained from the Harran University Experimental Animals Production and Research Centre and housed under standard specific pathogen-free (SPF) conditions at 22 ± 1 °C with a 12 h light/12 h dark cycle and free access to standard food and water.

The experimental diabetes model was induced by intraperitoneal administration of streptozotocin (STZ, 45 mg/kg). At 48 h after STZ administration, blood glucose levels were measured using blood samples collected from the tail vein, and animals with blood glucose concentrations ≥ 300 mg/dL were considered diabetic and included in the study. Following confirmation of diabetes, full-thickness excisional skin wounds (6 mm in diameter) were created in the foot region under general anesthesia. Subsequently, each wound was inoculated with 20 µL of an MRSA suspension (1 × 10^8^ CFU/mL) to establish the MRSA-infected diabetic wound model [33].

The infection was allowed to develop for 48 h before initiation of the corresponding treatments. The experimental animals were randomly divided into four groups (*n* = 5 per group): Mf-NE, NE, Fucidin, and an untreated MRSA-infected diabetic control group (STZ/MRSA). To minimize potential bias, the study was conducted in a blinded manner. Group allocation information was known only to the corresponding author, who maintained the allocation code throughout the study. The investigators involved in the conduct of the experiment, outcome assessment, and data analysis remained blinded to the group assignments until completion of the analyses. The allocation code was revealed only after the experimental evaluations and data analyses had been completed. Throughout the experimental period, the respective treatments were applied topically to the wound areas every other day, with NE and Mf-NE administered at a dose of 25 µL (for Mf-NE, approximately 1.25 mg of *M. fruticosa* essential oil per application, calculated from the 5% (*w*/*w*) loading and an approximate formulation density of 1 g/mL), while Fucidin was applied according to its standard treatment regimen. To monitor wound healing, wound areas were digitally photographed on days 0, 4, 8, and 12, and the measured wound areas were recorded for macroscopic evaluation. The remaining wound area was calculated as the wound area measured on each day relative to the initial wound area.

At the end of the experimental period (day 12), wound tissues were collected for histopathological, immunohistochemical, and microbiological analyses [33].

Remaining wound area (%) = S_n_/S_0_ × 100% (1)

where S_0_ was the original wound area, and S_n_ was the wound area at different times.

#### 2.10.2. Evaluation of In Vivo Antibacterial Efficacy

The antibacterial efficacy of the applied treatments at the wound site was evaluated using the colony-forming unit (CFU) counting method in order to determine the viable bacterial load. For this purpose, tissue samples were taken from the wound site of each group on day 12 of treatment. The samples were suspended in sterile phosphate-buffered saline (PBS) and homogenized. The tissue homogenates were serially diluted and spread onto Mueller–Hinton Agar. After the plates had been incubated at 37 °C for 18–24 h, the bacterial colonies that developed were counted and the colony-forming unit (CFU) values were calculated. Using these results, the bacterial loads at the wound sites of the treatment groups were compared and the in vivo antibacterial efficacy of the applications was evaluated [34].

#### 2.10.3. Histopathological and Immunohistochemical Examination

At the end of the experiment (day 12), wound tissue samples were fixed in 10% buffered formalin, processed routinely and embedded in paraffin. Sections of 5 µm thickness were cut and mounted on plain and adhesive slides. Sections mounted on plain slides were deparaffinized, rehydrated and stained with hematoxylin and eosin.

For immunohistochemistry, sections mounted on adhesive slides were deparaffinized and rehydrated. Endogenous peroxidase activity was quenched with 3% H_2_O_2_ and antigen retrieval was carried out in citrate buffer. After blocking of non-specific binding, primary antibodies against TNF-α and IL-1β were applied, followed by a biotinylated secondary antibody and streptavidin-peroxidase. Diaminobenzidine was used as the chromogen and Mayer’s hematoxylin as the counterstain. Sections were examined under a light microscope (Olympus BX53, Tokyo, Japan).

Erosive–ulcerative lesions, degenerative–necrotic changes, inflammatory cell infiltrates and vascular disturbance, together with TNF-α and IL-1β immunoreactivity, were scored semi-quantitatively as absent (−), mild (+), moderate (++) or severe (+++) according to severity.

#### 2.10.4. Gene Expression Analysis

Gene expression of TNF-α and IL-1β in the wound tissue was also examined by real-time PCR, with β-actin used as the housekeeping gene. The excised tissues were stored at −80 °C until the day of analysis. mRNA was isolated from the samples using a High Pure RNA isolation kit (12033674001 High Pure RNA Tissue Kit, Roche, Mannheim, Germany), following the manufacturer’s protocol [35]. RNA concentrations were equalized to 200 ng/µL. Total RNA was used for cDNA synthesis with the OneScript Plus cDNA Synthesis Kit (ABM, Richmond, BC, Canada) according to the commercial protocol, and cDNA yield was quantified by NanoDrop (Wilmington, DE, USA). Real-time PCR reactions were prepared in a total volume of 25 µL, containing 12.5 µL LightCycler^®^ FastStart DNA Master SYBR Green I (Roche, Mannheim, Germany), 2 µL cDNA, 2 µL of the forward and reverse primers, and ddH2O (DNase/RNase-free). Reactions were run on a real-time PCR instrument (Roche LC96, Mannheim, Germany) in three stages: denaturation (95 °C, 10 min); a three-step amplification program (95 °C for 10 s, primer annealing at the optimal temperature for 30 s, 72 °C for 10 s) for 45 cycles; and melting curve analysis (95 °C for 10 s, 65 °C for 60 s, 97 °C for 1 s, continuous mode). Relative expression of the target genes was normalized to β-actin and calculated using the 2^−ΔΔCt^ method. Primer sequences are listed in Table 1.

### 2.11. Statistical Analysis

For each animal a composite histopathological score was obtained by summing the grades for erosive–ulcerative lesions, degenerative–necrotic changes, inflammatory cell infiltrates and vascular disturbance (range 0–12), and a composite immunohistochemical score by summing the TNF-α and IL-1β grades (range 0–6). Because the grades are ordinal and each group contained five animals, the groups were compared using the Kruskal–Wallis test followed by Dunn’s post hoc test with Bonferroni correction. Effect sizes are given as epsilon-squared (ε^2^ = H/(n − 1)) for the Kruskal–Wallis test and as Cliff’s delta for pairwise comparisons. Every significant comparison was recalculated with each animal excluded in turn, to establish whether it depended on a single animal. Two-sided *p* values below 0.05 were considered significant. Analyses were carried out in R 4.5.3.

## 3. Results and Discussion

### 3.1. Content and Chemical Composition of Essential Oils of M. fruticosa

**Figure 1:**
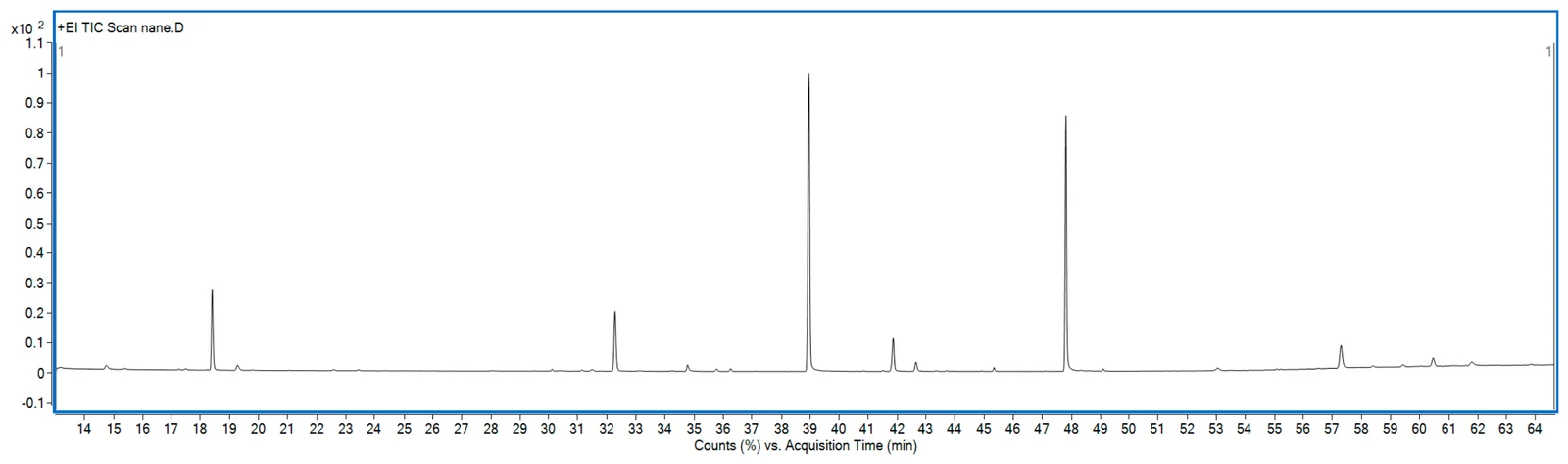
Total ion chromatogram (TIC) of *Micromeria fruticosa* essential oil obtained by GC–MS analysis.

The chemical profile of *M. fruticosa* essential oil was determined by GC–MS analysis (Figure 1), leading to the identification of 12 constituents representing 100% of the total oil composition (Table 2).

This compositional pattern is consistent with previous studies, which report *M. fruticosa* essential oil as being rich in monoterpene derivatives, particularly oxygenated forms [38,39]. Pulegone (42.81%) was identified as the major component, followed by piperitenone (29.73%) and isomenthone (9.40%). Other major constituents included piperitone (5.38%), hexadecane (4.75%), 1-hexadecanol, 2-methyl- (1.77%) and bicyclogermacrene (1.59%).

In contrast, monoterpene hydrocarbons such as β-pinene (0.93%) and D-limonene (1.05%) were present only in low amounts. Oxygenated monoterpenes constituted the dominant fraction of the essential oil, with pulegone (42.81%) and piperitenone (29.73%) being the major constituents. These compounds are widely recognized as characteristic metabolites of several *Micromeria* species and contribute significantly to their aroma profile and biological activities.

The quality and potential industrial value of *Micromeria* essential oil are largely determined by its chemical composition, particularly the relative abundance of oxygenated monoterpenes. Essential oils rich in pulegone and piperitenone have attracted considerable interest due to their antimicrobial, antioxidant, and aromatic properties reported in previous studies. In the present study, the high proportions of pulegone and piperitenone, together with notable amounts of isomenthone and piperitone, indicate the potential applicability of the oil in pharmaceutical, cosmetic, and fragrance industries.

Essential oils of *M. fruticosa* are commonly characterized by high concentrations of oxygenated monoterpenes, while sesquiterpenes and monoterpene hydrocarbons generally occur at lower levels. In the present study, oxygenated monoterpenes represented the predominant chemical class, accounting for the vast majority of the identified constituents, whereas sesquiterpene hydrocarbons were represented only by bicyclogermacrene (1.59%). However, the relative composition of these constituents may vary considerably depending on geographical origin, climatic conditions, phenological stage, and environmental factors. The predominance of pulegone and piperitenone is consistent with previous reports on several *Micromeria* species. Such variations in the proportion of individual compounds further emphasize the influence of ecological and technological factors on essential oil composition and highlight the importance of detailed chemical characterization for quality assessment and standardization.

### 3.2. Development of Micromeria Essential Oil Containing Nanoemulsion Formulations

#### 3.2.1. Pseudoternary Phase Diagrams

Pseudoternary phase diagrams play a critical role in nanoemulsion development by mapping the compositional domains where stable and homogeneous systems are obtained, thereby facilitating the optimization of oil, surfactant/cosurfactant, and aqueous phase ratios [40]. By providing a visual representation of the formulation space, the ternary phase diagram serves as a valuable tool for identifying the ideal ranges of components required to produce high-quality nanoemulsions with desired characteristics [22,41]. Consistent with our findings, Tartari et al. [41] used a pseudoternary phase diagram to identify the nanoemulsion region and optimize component ratios, demonstrating the importance of phase diagram analysis in the rational design of stable nanoemulsion systems.

**Figure 2:**
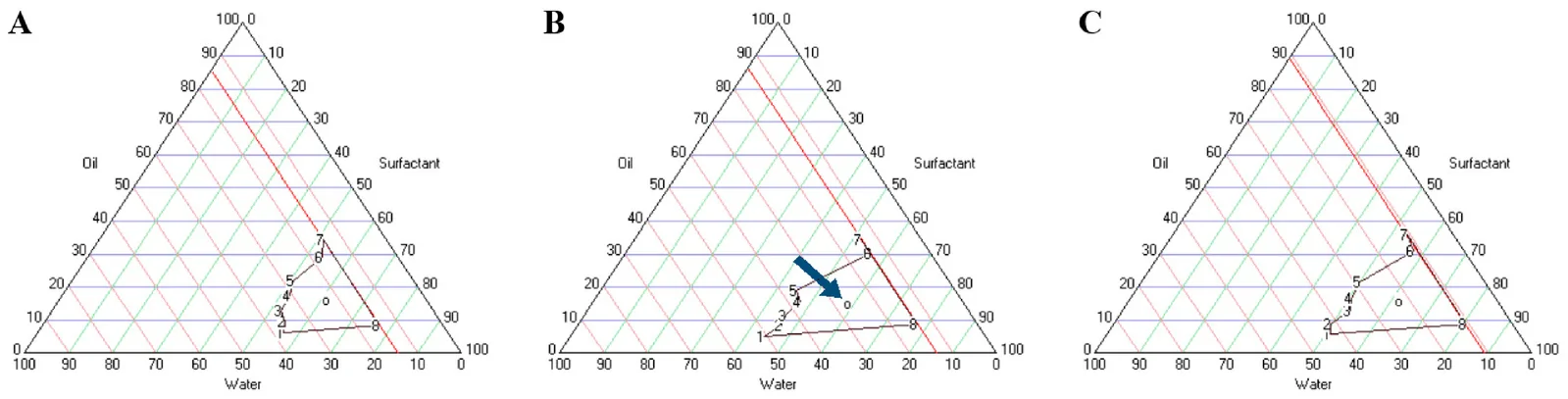
Pseudoternary phase diagrams of nanoemulsions (**A**) NE1:1; (**B**) NE2:1; (**C**) NE 3:1. The outlined area is the nanoemulsion region; the arrow in (**B**) marks the selected formulation (Table 3).

In all pseudoternary phase diagrams, the composition of the cosurfactant mixture was kept constant, whereas the surfactant to cosurfactant ratio was systematically varied. As shown in Figure 2, three different combinations were evaluated, with surfactant:cosurfactant ratios. Nanoemulsion formation is determined not only by the type of components used but also by their compositional ratios within the system. In such diagrams, the highlighted area indicates the region where nanoemulsions are formed, while other areas correspond to turbid or macroemulsion systems. An expanded nanoemulsion region suggests a higher potential for achieving stable and clear formulations [42]. Comparison of the pseudoternary phase diagrams demonstrated that the extent of the nanoemulsion region varied depending on the surfactant/cosurfactant ratio. Three different Smix ratios were evaluated: 1:1 (Figure 2A), 2:1 (Figure 2B), and 3:1 (Figure 2C). Among these, the largest nanoemulsion region was observed at the 2:1 surfactant/cosurfactant ratio. When the ratio was adjusted to 1:1, where the relative amount of surfactant was reduced, the nanoemulsion area became smaller. Similarly, increasing the surfactant proportion relative to the cosurfactant (3:1) also resulted in a reduction in the nanoemulsion region. These findings indicate that the nanoemulsion domain is strongly influenced by the balance between surfactant and cosurfactant concentrations. Based on these results, the 2:1 surfactant/cosurfactant ratio was selected as the optimal Smix composition, and a formulation located near the center of the nanoemulsion region in this phase diagram was chosen for further studies.

#### 3.2.2. Preparation of Nanoemulsion Formulations

The pseudoternary phase diagram constructed using oleic acid (oil phase), a Cremophor^®^ EL/Tween 80–Transcutol^®^ surfactant system, and distilled water revealed a distinct nanoemulsion region. Based on this diagram, the formulation located at the central part of the nanoemulsion domain, corresponding to a 2:1 surfactants/cosurfactant ratio, was selected as the placebo formulation. The active nanoemulsion was subsequently obtained by incorporating *Micromeria* essential oil into the oil phase at a concentration of 5% (*w*/*w*), while maintaining the same formulation approach used for the placebo system (Table 3). The 5% (*w*/*w*) essential oil was based on a balance between achieving sufficient biological activity and limiting the potential toxicity associated with higher essential oil concentrations. Although essential oils are naturally derived, increasing their concentration does not necessarily provide additional benefits and may increase concerns regarding cytotoxicity and dermal tolerability [43]. For this reason, lower effective essential oil concentrations are generally preferred in topical nanoemulsion systems. The selection of 5% (*w*/*w*) was further supported by previous reports demonstrating the suitability of this concentration for essential oil-loaded nanoemulsions [44,45,46]. Llinares et al. [44] reported that nanoemulsions containing 5 and 7.5% rosemary essential oil exhibited better physical stability, whereas increasing the essential oil concentration to 10% resulted in increased droplet size and polydispersity. Similarly, Cossetin et al. [45] successfully developed nanoemulsions containing 5% *Lavandula dentata* and *Myristica fragrans* essential oils with nanometric droplet sizes and favorable physicochemical characteristics. Therefore, 5% (*w*/*w*) was selected as an empirical concentration of *Micromeria fruticosa* essential oil for incorporation into the nanoemulsion formulation, rather than as an optimized concentration established through comparative biological activity or toxicity testing at different essential oil concentrations.

Oleic acid was selected as the oil phase because of its suitability for lipid-based formulations and its capacity to accommodate lipophilic constituents. Its selection was also supported by previous nanoemulsion studies demonstrating effective emulsification of oleic acid by Tween 80 [47,48]. In our previous studies on essential oil-loaded nanoemulsions, oleic acid was likewise used as the oil phase in combination with Cremophor^®^ EL [24] or a Cremophor^®^ EL/Tween 80 mixture [46], and physically stable nanoemulsions were obtained. Tween 80 was selected as a non-ionic, high-HLB surfactant because polysorbates, particularly Tween 80, are among the most widely used surfactants for essential oil-based colloidal systems and have demonstrated favorable emulsification efficiency and the ability to produce small and stable droplets. Cremophor^®^ EL was combined with Tween 80 to further improve emulsification and interfacial stabilization. Previous studies on essential oil nanoemulsions have shown that Cremophor^®^ EL exhibits strong emulsifying properties, comparable or even superior to those of Tween 80 in some systems and can form a stable interfacial film at relatively low concentrations. Transcutol^®^ was employed as the co-surfactant because of its amphiphilic character and its ability to increase interfacial film flexibility and further reduce interfacial tension, thereby facilitating nanoemulsion formation [43,49]. Oleic acid is also an established permeation enhancer and is widely used as the oil phase of dermal micro- and nanoemulsions together with Transcutol^®^, which has been reported to increase drug solubility in such systems [40]. Accordingly, the oleic acid–Cremophor^®^ EL/Tween 80–Transcutol^®^ system was selected to provide an appropriate oil–surfactant–co-surfactant environment for incorporating the lipophilic constituents of *M. fruticosa* essential oil into an aqueous nanoemulsion. Cremophor^®^ EL was used at a concentration of 19.36% (*w*/*w*) in the present formulation. Although surfactant tolerability is concentration- and route-dependent, Cremophor^®^ EL has been successfully employed in formulations intended for topical application. Notably, da Silva et al. [50] developed an essential oil-based nanoemulsion containing 30% (*v*/*v*) Cremophor^®^ EL, which was classified as nonirritating in the HET-CAM irritation assay. These findings, together with previous reports of Cremophor^®^ EL containing topical nanoemulsions, support the suitability of its use at the concentration employed in the present formulation.

### 3.3. Characterization of Nanoemulsion Formulations

#### 3.3.1. Droplet Size and Size Distribution

The droplet size and size distribution obtained in the present study are in line with previous findings highlighting the importance of formulation-related parameters in nanoemulsion systems. In particular, surfactant type and concentration play a main role in reducing interfacial tension and stabilizing oil droplets, thereby leading to smaller droplet sizes and narrower size distributions. Campolo et al. [51] demonstrated that optimized surfactant systems yield nanoemulsions with improved homogeneity and reduced polydispersity, depending on the characteristics of the oil phase. Similarly, the droplet size range and low PDI values observed in our formulations indicate that the selected surfactant composition and ratio were effective in producing physically stable oil-in-water nanoemulsions.

The mean droplet size of the blank nanoemulsion was 214.5 ± 14.4 nm with a PDI of 0.402 ± 0.01. Following the incorporation of 5% (*w*/*w*) *Micromeria* essential oil, the mean droplet size increased to 292.9 ± 9.9 nm, accompanied by an increase in the PDI to 0.560 ± 0.01 (Table 4). The gradual increase in droplet size and PDI observed with increasing *Micromeria* essential oil concentration may be attributed to alterations in the oil to surfactant ratio and the limited ability of the surfactant system to fully stabilize higher oil loads. A similar trend was reported by Hassanzadeh et al. [52], who demonstrated that increasing garlic essential oil content led to larger droplet sizes and broader size distributions, which were associated with insufficient interfacial coverage at elevated oil concentrations. Consistent with these findings, the incorporation of *Micromeria* essential oil into the blank nanoemulsion resulted in an increase in both droplet size and PDI, indicating that the presence of the essential oil influenced droplet characteristics and nanoemulsion homogeneity, while the formulation remained within the nanoscale size range [53].

#### 3.3.2. Zeta Potential

Zeta potential is widely employed as an indicator of colloidal behavior in nanoemulsions and related dispersed systems, as it reflects the magnitude of interparticle interactions governing droplet association or separation. In general, higher absolute zeta potential values correspond to stronger electrostatic repulsive forces, thereby decreasing the likelihood of droplet aggregation. Conversely, formulations exhibiting low surface charge may display limited electrostatic stabilization. Nevertheless, it is well established that nanoemulsions stabilized with nonionic surfactants or polymeric agents can maintain physical stability predominantly through steric hindrance rather than electrostatic repulsion. In such cases, stable dispersion can be achieved even when zeta potential values remain close to neutrality, indicating that colloidal stability is preserved despite relatively low surface charge [22,54]. The zeta potential values of both the blank and 5% *Micromeria* essential oil-loaded nanoemulsions were approximately neutral, remaining close to 0 mV (Table 4). The zeta potential values of the formulations are presented in Table 4. The blank nanoemulsion exhibited a zeta potential of 0.0 ± 0.1 mV. Similarly, nanoemulsion 5% (*w*/*w*) *Micromeria* essential oil showed zeta potential values of 0.0 ± 0.1 mV, indicating no measurable difference in surface charge among the formulations [55,56].

Overall, the PDI and zeta potential findings should be interpreted in the context of the physicochemical characteristics and stabilization mechanism of the nanoemulsion system. The PDI values were 0.402 ± 0.01 for the blank NE and 0.560 ± 0.01 for Mf-NE, indicating a moderately polydisperse droplet population. Comparable PDI values have been reported for nanoemulsion systems that maintained physical stability during storage [57]. Both formulations exhibited a near-neutral zeta potential (0.0 ± 0.1 mV). However, a low absolute zeta potential does not necessarily indicate instability in systems stabilized by nonionic surfactants, since steric stabilization may predominate over electrostatic repulsion. In the present formulation, Tween 80 and Cremophor^®^ EL can provide such steric stabilization through their hydrophilic chains at the oil–water interface. Indeed, nanoemulsions with zeta potentials close to 0 mV have previously been reported to maintain stability during prolonged storage, supporting the importance of considering the stabilization mechanism rather than zeta potential alone [54,57]. However, these comparisons do not establish storage stability for the present formulations. A near-neutral zeta potential alone neither proves stability nor demonstrates instability; longitudinal measurements of droplet size, PDI and physical changes are required to evaluate storage stability.

#### 3.3.3. Conductivity

Electrical conductivity measurements provide valuable information regarding the nature of the continuous phase in nanoemulsion systems. Since ionic movement is facilitated in aqueous environments, higher conductivity values are typically associated with oil-in-water systems, whereas formulations with an oil continuous phase generally exhibit negligible conductivity. In this study, all tested formulations showed conductivity values close to 0.2 mS/cm, supporting the presence of an aqueous continuous phase with the oil phase confined within the internal droplets. Importantly, the addition of the essential oil did not result in any noticeable variation in conductivity, indicating that the structural integrity of the oil-in-water system was preserved and no phase inversion occurred. These observations agree with previously published findings, and the relevant conductivity data are presented in Table 4 [58].

#### 3.3.4. pH

In topical wound healing cream and gel formulations, pH is a critical parameter influencing both skin compatibility and the wound healing process. Therefore, the pH of the formulation should be maintained within a dermatologically acceptable range and, when possible, adjusted toward a mildly acidic environment [59]. The pH values of the formulations are summarized in Table 5. The blank nanoemulsion exhibited a pH of 5.44 ± 0.1. Nanoemulsions containing *Micromeria* essential oil showed comparable pH value, measured as 5.92 ± 0.1 for the formulation containing 5% (*w*/*w*) essential oil.

The skin surface is characterized by a mildly acidic milieu, typically within a 4–6 pH range, and plays an indispensable part in maintaining epidermal barrier function, organizing lipid structures, controlling cutaneous microbiota, and modulating the inflammatory response. Formulations adjusted to this range can maintain barrier integrity and support dermatological health, while topical formulations with pH values outside this physiological interval may disturb homeostasis of the skin. Previously, it has been suggested in the literature that topical formulations should be developed with pH between 4.0 and 6.0. Within this context the pH profiles of the formulations suggest a favorable compatibility with topical application as well as low risk for irritation due to changes in pH, and possible benefits for skin barrier maintenance and microbalances [60,61].

#### 3.3.5. Viscosity and Rheological Measurement

Viscosity and rheological properties are key parameters in the development of topical diabetic wound healing formulations as they directly affect application behavior, spreadability, site retention, and patient compliance. The flow characteristics of a formulation influence its capacity to be applied with ease under shear and remain on the wound surface after application owing to sufficient structural integrity [62,63]. Therefore, detailed rheological evaluation is essential to assess the suitability of nanoemulsion systems for topical wound management.

The apparent viscosity values of the formulations are presented in Table 5. The blank nanoemulsion exhibited a low viscosity of 42.33 ± 2.7 mPa·s. Similarly, nanoemulsions containing *Micromeria* essential oil showed comparably low viscosity values, measured as 29.49 ± 1.6 mPa·s for formulation containing 5% (*w*/*w*) essential oil [54,64,65].

According to Pavoni et al. [43], formulation parameters such as the nature of the oil phase, surfactant concentration and dispersed phase affect nanoemulsions viscosity. The inclusion of essential oil from *Micromeria* 5% in present research reduced the viscosity from 42.33 to 29.49 mPa·s implicating that the flow properties of nanoemulsion were modified while retaining characteristics typical for low viscosity of nanoemulsion systems.

**Figure 3:**
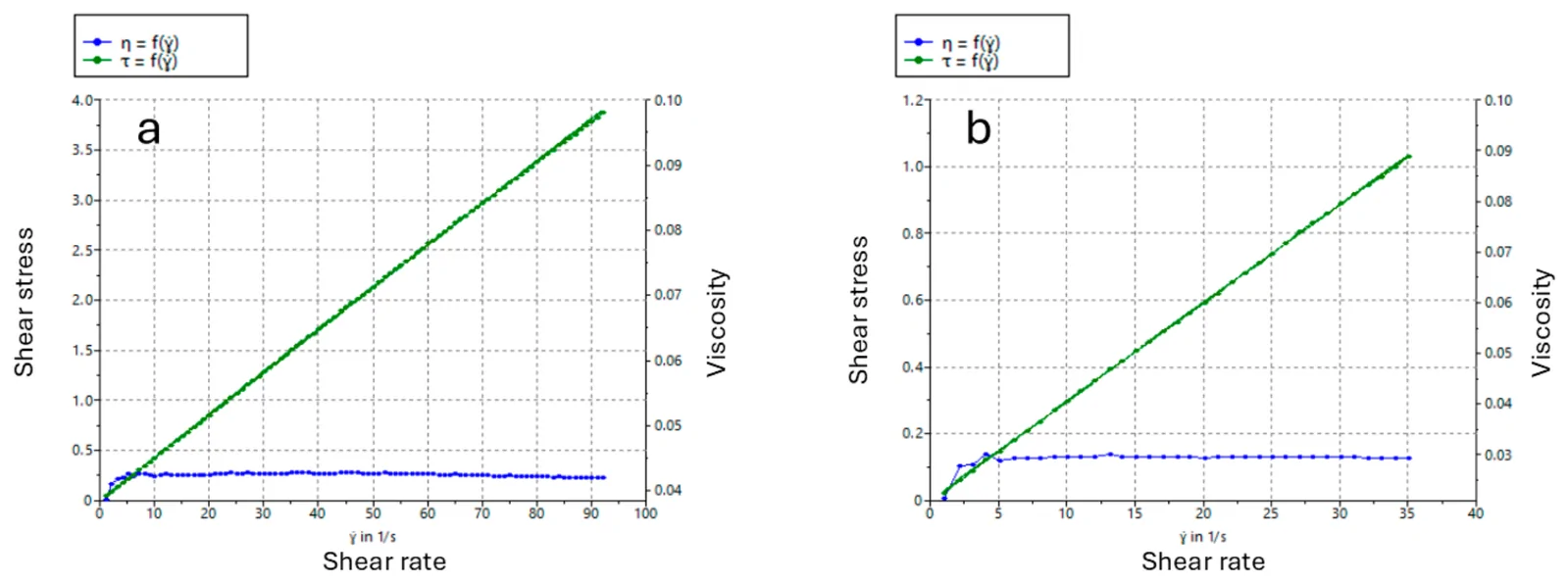
Rheograms showing the relationship between shear rate and shear stress. (**a**) NE2:1 (Blank), (**b**) NE2:1 (5% NE).

According to Newton’s law of viscosity, Newtonian fluids exhibit a linear relationship between shear stress and shear rate, whereas non-Newtonian fluids require nonlinear constitutive equations to describe their rheological behavior [66]. The linear increase in shear stress with increasing shear rate and the constant apparent viscosity throughout the investigated shear rate range indicate typical Newtonian flow behavior for both the blank and *Micromeria* essential oil loaded nanoemulsions (Figure 3). These findings agree with those reported by Vats and Kumar [67], who demonstrated that ultrasound-assisted oil-in-water nanoemulsions exhibited Newtonian flow behavior with a linear shear stress–shear rate relationship and shear rate-independent viscosity. Additionally, the nanoemulsion containing the essential oil showed lower apparent viscosity compared to the blank formulation, indicating that incorporation of *Micromeria* essential oil reduced flow resistance through this nanocarrier system without modifying its rheological properties. Such Newtonian flow behavior is advantageous for topical nanoemulsions so that they are smoothly spread when applied and have good predictability in flow behavior during processing and handling.

### 3.4. Evaluation of Microbiological Assays

**Figure 4:**
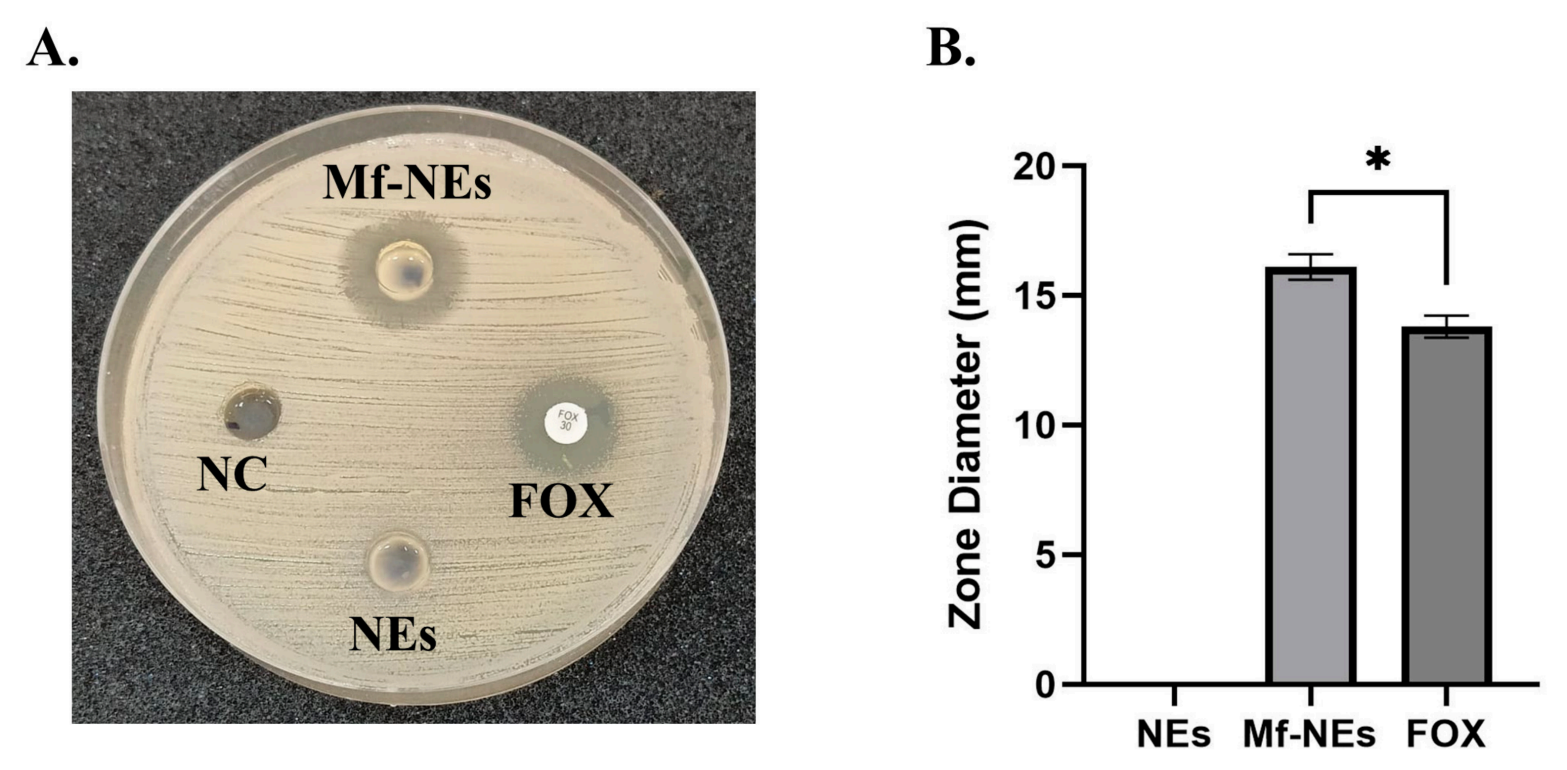
Inhibition zone diameters (mm) of Mf-NE and NE determined by the agar well diffusion method. (**A**) Overall inhibition zones. (**B**) Antibacterial activity against MRSA. Data are presented as mean ± SD (*n* = 3). * *p* < 0.05 was considered statistically significant.

The antibacterial activity of the nanoemulsion formulations against the MRSA strain is presented in Figure 4. The NE formulation did not produce any inhibition zone. In contrast, the Mf-NE formulation produced an inhibition zone 16.1 mm in diameter, demonstrating marked antibacterial activity. The inhibition zone produced by the cefoxitin disk used as a positive control was measured as 13.8 mm. According to the microdilution method performed with the resazurin assay, the MIC value of the Mf-NE against the MRSA strain was determined as 1.25 mg/mL, whereas the MIC value of the NE was determined as 5 mg/mL.

Essential oils are reported to possess a range of biological activities, most notably antioxidant, anti-inflammatory and antibacterial effects [68,69]. The MIC values of *M. fruticosa* subsp. *serpyllifolia* essential oil against *S. aureus* and methicillin-resistant MRSA strains have been reported to lie in the range of 3.125–6.250 mg/mL [13]. In another study, pollen extracts obtained from *M. fruticosa* showed an MIC value of 5 mg/mL against *S. aureus* [15].

Research conducted in recent years has focused on revealing the antibacterial potential of essential oil (EO)-based nanoemulsions. These studies have reported that such formulations exhibit effective antibacterial activity against clinically and food-borne important pathogens such as *Salmonella enterica*, *Escherichia coli*, *Pseudomonas aeruginosa*, *S. aureus*, *Salmonella typhimurium*, *Listeria monocytogenes*, *Klebsiella oxytoca* and *Acinetobacter baumannii* [54,70,71,72,73]. In a study conducted by Hassan and Mujtaba (2019), the antibacterial efficacy of nanoemulsions containing *Allium sativum* essential oil was investigated. The findings showed that the developed nanoemulsions exerted a stronger antibacterial effect on *Staphylococcus aureus* than on *Escherichia coli* [74]. Kayıran et al. found MIC values ranging from 12.5 to 50 mg for nanoemulsion forms containing *C. citratus* essential oil against the MRSA strain. In their study, inhibition zones ranging from 20.67 to 41.67 mm were observed against the same strain [46].

Direct topical application of *M. fruticosa* essential oil may cause irritation and other undesirable reactions on the skin. For this reason, nanoemulsion systems are regarded as safer and more suitable carrier systems for topical use. In nanoemulsions, the active components are distributed in a balanced manner between the oil and water phases according to their solubility characteristics, which supports both the stability of the formulation and the delivery of the active substance. In addition, owing to their natural bioactive effects, nanoemulsions strengthen the skin barrier function by reducing trans-epidermal water loss. Because of these properties, nanoemulsions are regarded as an effective approach for incorporating plant-derived bioactive compounds into formulations [75].

### 3.5. Antioxidant Activity

**Figure 5:**
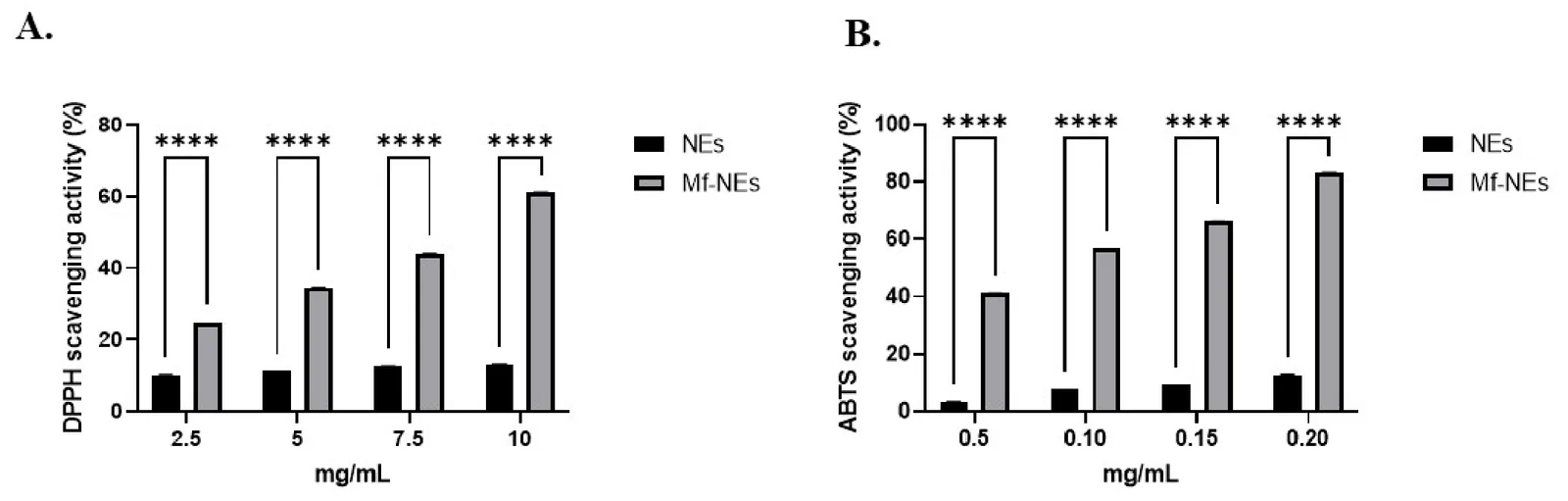
Antioxidant activity of NE and Mf-NE formulations. (**A**) DPPH, (**B**) ABTS. Data are presented as mean ± SD (*n* = 3). **** *p* < 0.0001 was considered statistically significant.

The free radical scavenging capacities of the NE and Mf-NE formulations were evaluated at different concentrations by DPPH and ABTS analyses. The Mf-NE formulation was found to exhibit marked antioxidant activity owing to the polyphenolic compounds present in the *M. fruticosa* essential oil it contains [76]. The radical scavenging capacity of the Mf-NE increased in a concentration-dependent manner, and the inhibition values obtained by the ABTS method were found to be higher than those obtained by the DPPH method. In the DPPH analysis, the highest inhibition rate was determined as 61.5% at 10 mg/mL. In the ABTS analysis, the radical scavenging rate was found to be 71.5% at 0.2 mg/mL. In contrast, the blank nanoemulsion (NE) formulation showed low antioxidant activity at the same concentrations, with a DPPH radical scavenging activity of 13.2% and an ABTS radical scavenging activity of 12.8% (Figure 5). These findings indicate that the *M. fruticosa* essential oil retains its antioxidant properties within the nanoemulsion system and that the Mf-NE formulation has the potential to be considered a natural antioxidant agent.

Phenolic compounds have been identified as the main constituents responsible for the antioxidant activity of plant-derived antioxidants. The antioxidant effect of these compounds arises from their redox-based properties, such as their ability to donate electrons or hydrogen, to quench singlet oxygen and to chelate metal ions [77,78]. The findings obtained in our study are consistent with previous research on nanoemulsion formulations of different essential oils and the isolated compounds derived from them. Lou et al. reported that the nanoemulsion form of *Citrus medica* essential oil exhibited higher DPPH radical scavenging activity than the free essential oil. The researchers determined the DPPH radical inhibition rates as 44.3% for the essential oil and 72.4% for the nanoemulsion form [79]. Similarly, Zamaniahari et al. reported that the nanoemulsion form of *Mentha spicata* L. essential oil showed stronger antioxidant activity than the non-nanoemulsified form [80]. These antioxidant effects provide a more efficient electron transfer mechanism than the non-nanoemulsified versions by facilitating the contact of bioactive compounds with free radicals [81].

### 3.6. Evaluation of Biocompatibility

**Figure 6:**
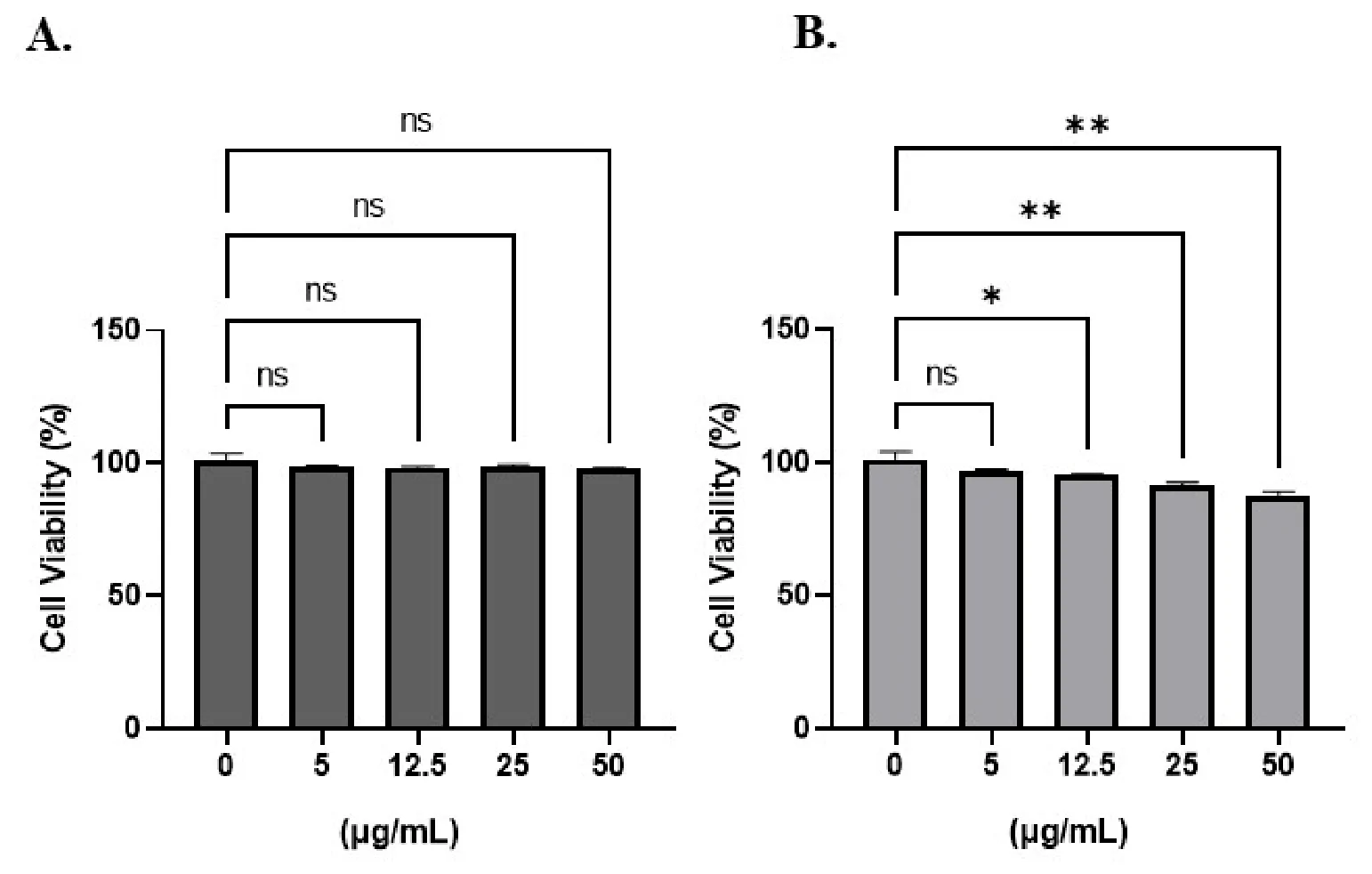
Effects of NE (**A**) and Mf-NE (**B**) formulations on cell viability of L929 fibroblasts assessed by MTT assay (*n* = 3). ns: not significant, * *p* < 0.05, ** *p* < 0.01 represents a significant difference between control and individual treatments.

The effects of NE and Mf-NE on the viability of L929 cells were investigated, with the corresponding cell viability percentages presented in Figure 6. The viability of cells remained above 87% regardless of the tested concentration, indicating good cytocompatibility of all formulations under the experimental conditions. For the cytocompatibility assessment, concentrations ranging from 0–50 μg/mL were selected to represent a biologically relevant in vitro exposure range [82,83]. However, the concentrations applied under in vitro conditions should not be considered directly equivalent to the topical doses used in vivo. The actual cellular exposure within living tissue may be influenced by several factors, including the penetration capacity of the nanoformulation, dilution by surrounding biological fluids, and alterations in the barrier properties of infected tissue. Therefore, the present in vitro findings should be interpreted as supportive evidence of the cellular compatibility of the formulations rather than as a direct representation of the actual exposure levels achieved in vivo. The MTT results cannot be extrapolated directly to the undiluted topical formulation, local irritation or systemic pulegone exposure.

Pulegone accounted for 42.81% of the normalized GC–MS peak area of the essential oil. Combining this relative abundance with the 5% (*w*/*w*) oil loading gives an approximate nominal pulegone content of 2.14% (0.05 × 42.81%). This estimate assumes that normalized peak area approximates mass fraction; it is not a validated measurement of pulegone mass in the formulation or of the absorbed dose. The applied volume of 25 μL per wound therefore cannot by itself establish systemic exposure or a safety margin.

The EMA public statement identifies daily limits for combined pulegone and menthofuran in cutaneous herbal medicines of 37.5 mg/adult for lifelong use and 75 mg/adult for use shorter than one year, with cutaneous use restricted to intact skin [84]. These adult limits cannot establish safety in a rat open-wound model. The amended cosmetic assessment of peppermint-derived ingredients removed the earlier 1% pulegone restriction, but its conclusion concerns specified cosmetic uses formulated to be non-sensitizing, not this *Micromeria* formulation [85]. Neither assessment provides a validated safe concentration for the present treatment of compromised diabetic skin.

Accordingly, the current findings support preclinical efficacy and assay-specific cytocompatibility, but do not establish local or systemic safety of the topical regimen. Formulation-specific studies of irritation, sensitization, dermal absorption and systemic exposure, including liver safety endpoints and dose–response evaluation, are needed before a safety margin or clinical use can be justified.

### 3.7. In Vivo Studies

#### 3.7.1. Evaluation of Wound Healing in MRSA-Infected Diabetic Foot Wounds

In this study, an MRSA-infected diabetic foot wound model was established in order to evaluate the therapeutic efficacy of the NE and Mf-NE formulations. The infected wounds were treated for 12 days with the formulations assigned to the experimental groups, and wound healing was assessed macroscopically at the specified time points.

**Figure 7:**
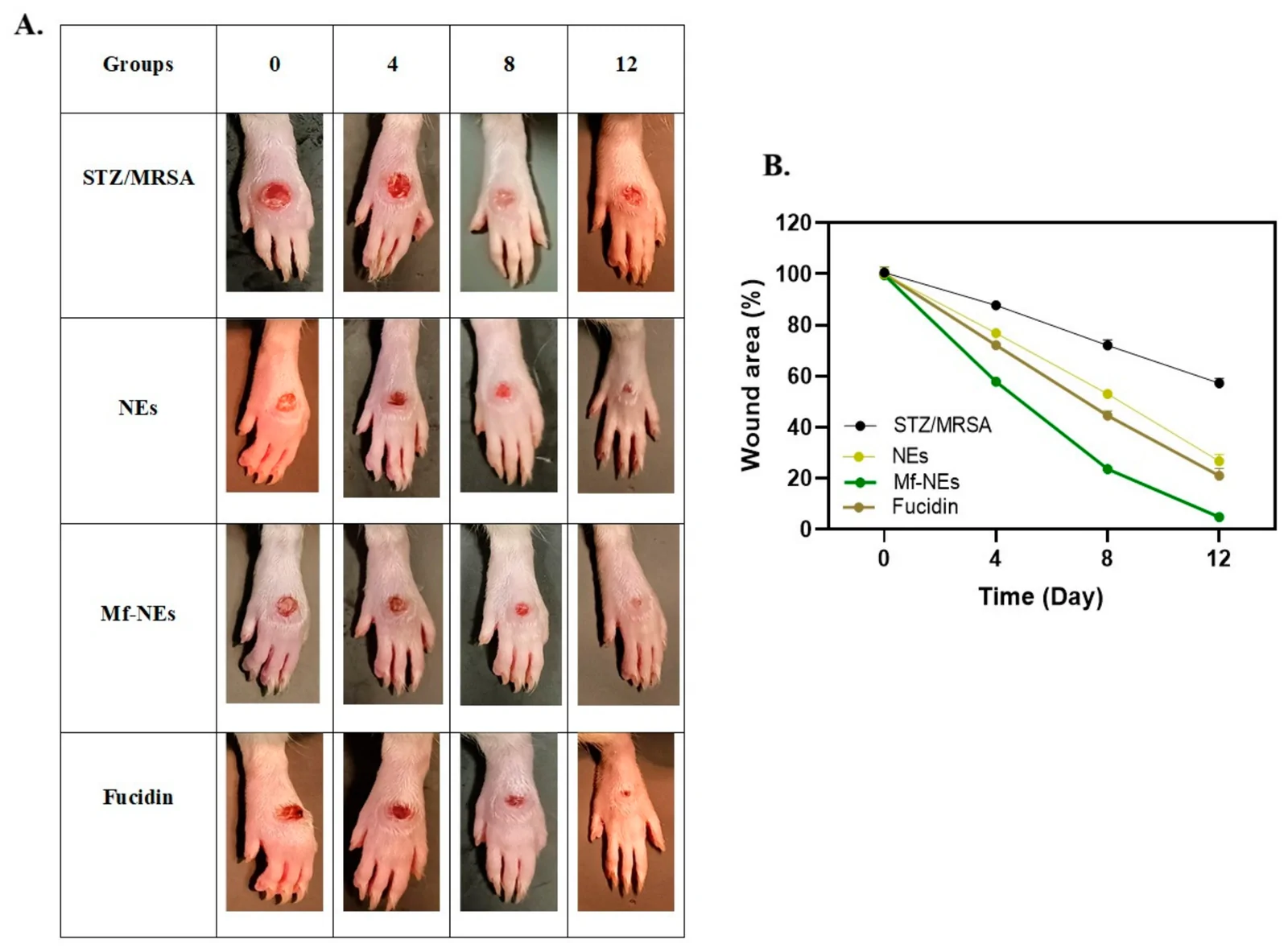
In vivo wound healing evaluation of different NE and Mf-NE formulations. (**A**) Representative photographs of full-thickness excisional wounds in the untreated STZ/MRSA, NE, Mf-NE, and Fucidin-treated groups (*n* = 5 per group) at days 0, 4, 8, and 12 post-wounding. (**B**) Remaining wound area (%) relative to the initial wound area on day 0 over the 12-day treatment period; smaller values indicate greater closure. Data demonstrate a time-dependent reduction in wound area, with NE, Mf-NE and Fucidin-treated groups showing enhanced wound healing compared to the STZ/MRSA group. Data are presented as mean ± SD (*n* = 5 per group).

The effect of the prepared nanoformulations on wound healing was evaluated comparatively between the experimental groups. Wound areas were measured on days 4, 8 and 12, and the results obtained are shown in Figure 7. Throughout the treatment period, the wound area was observed to decrease over time in all groups. However, the lowest healing was observed in the STZ/MRSA group, and at the end of day 12, the remaining wound area was calculated as 57.2 ± 1.3%. A greater reduction in wound area was observed in the group treated with NE compared with the STZ/MRSA group, and the remaining wound area was determined as 26.1 ± 1.9%. In the group treated with Mf-NE, wound healing was found to be markedly accelerated. At the end of the twelfth day, the remaining wound area in the Mf-NE-treated group was calculated as 4.8 ± 0.9%. In the Fucidin group, which was used as a positive control, the remaining wound area was determined as 21.0 ± 1.9%. Thus, the 4.8 ± 0.9% remaining area in the Mf-NE group corresponds arithmetically to 95.2 ± 0.9% wound closure; throughout this manuscript, the measured outcome is reported as remaining wound area.

Although the NE formulation did not produce a detectable inhibition zone in the agar diffusion assay, its MIC value of 5 mg/mL and the marked reduction in bacterial density observed in the NE-treated group indicate its potential antibacterial activity. The absence of an inhibition zone may be related to the limited diffusion of nanoemulsion droplets through the agar matrix. In vivo, the prolonged contact and local retention of the nanoemulsion at the infected wound site may enhance its interaction with bacterial cells. Moreover, the nanoscale droplet size and large interfacial area of nanoemulsions may improve the local availability of active compounds and facilitate interactions with bacterial membranes, thereby contributing to bacterial reduction and wound healing [86,87,88].

Factors such as the excessive production of reactive oxygen species (ROS) in infected wounds, an intense inflammatory response and dysfunction of immune cells adversely affect the wound healing process. For this reason, current research is concentrating on the development of treatment strategies that bring infection under control while at the same time supporting tissue repair and wound healing, rather than focusing solely on reducing the microbial load [89,90]. In one study, evaluation of in vivo wound healing efficacy revealed a marked reduction in wound size following treatment with selected cetrimide-loaded nanoemulsions [91]. In another study, the topical antimicrobial NB-201 was effective in significantly alleviating inflammation and supporting wound healing in treated MRSA-infected burn wounds [92]. Our study demonstrated that the Mf-NE formulation in particular reduced the wound area more effectively and showed superior wound healing activity compared with all other groups. The acceleration of wound closure and the more effective re-formation of epithelial tissue may be attributable to the NE formulation increasing the bioavailability of the active component in its content by retaining it at the wound site for a longer period [93].

#### 3.7.2. In Vivo Antibacterial Efficacy

**Figure 8:**
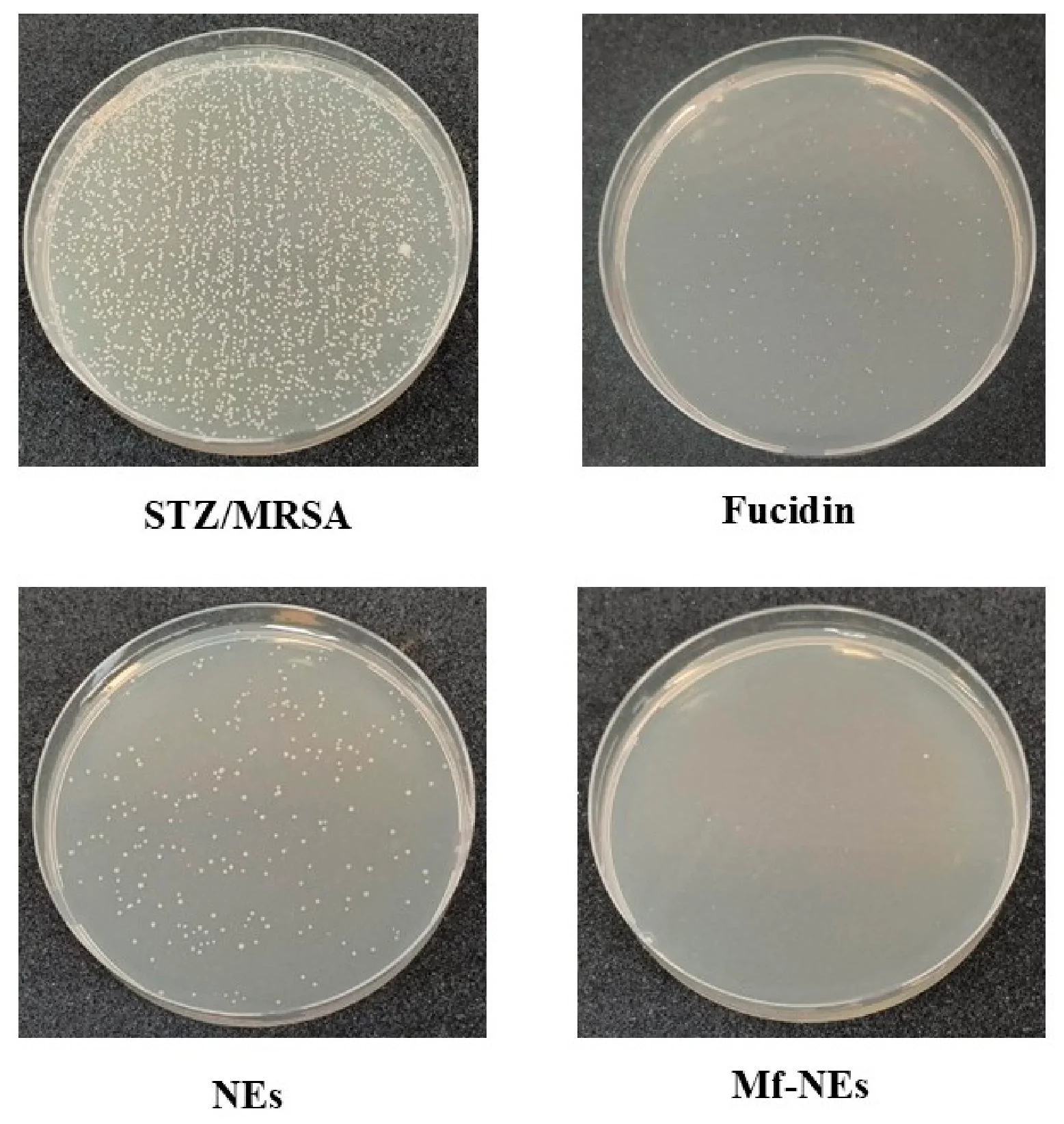
Representative photographs of bacterial colonies isolated from wound sites on day 12 following treatment.

In our study, antibacterial efficacy was evaluated using wound samples collected on day 12 of treatment in MRSA-infected diabetic foot wounds. On day 12 of treatment, the highest bacterial load was observed in the untreated STZ/MRSA group in the tissue samples taken from the MRSA-infected diabetic foot wounds. In contrast, a marked reduction in bacterial density was observed in the groups treated with Fucidin and NE. In particular, the almost complete suppression of MRSA colonization in the wounds treated with Mf-NE demonstrated superior antibacterial efficacy compared with the untreated STZ/MRSA group (Figure 8).

Bacterial infections are among the most important factors that adversely affect wound healing. The proliferation of microorganisms at the wound site may lead to prolongation of the inflammatory process, an increase in the amount of exudate, damage to the tissue structure, an increased risk of developing systemic infection and increased scar formation. For this reason, the effective prevention and control of wound infections is of critical importance for healthy tissue repair to take place. This situation reveals the need to develop and use wound dressings with high antimicrobial efficacy [94]. Cao et al. showed that NB-201 treatment significantly reduced MRSA colonization in infected burn wounds [92]. In another study, a chlorhexidine acetate nanoemulsion was shown to be the most effective agent tested against MRSA infection in skin burn wounds [95]. Similar results were obtained in our study, and the antimicrobial efficacy of the applied nanoemulsion formulation against MRSA supports the previous literature. This similarity may be explained by nanoemulsion systems increasing the bioavailability of the active substance, interacting more effectively with the bacterial cell membrane and enhancing antimicrobial efficacy in tissues predisposed to diabetic infection. Therefore, our findings show that nanoemulsion-based approaches may be a promising option in the treatment of MRSA-infected diabetic foot wounds.

#### 3.7.3. Histopathological Findings

**Figure 9:**
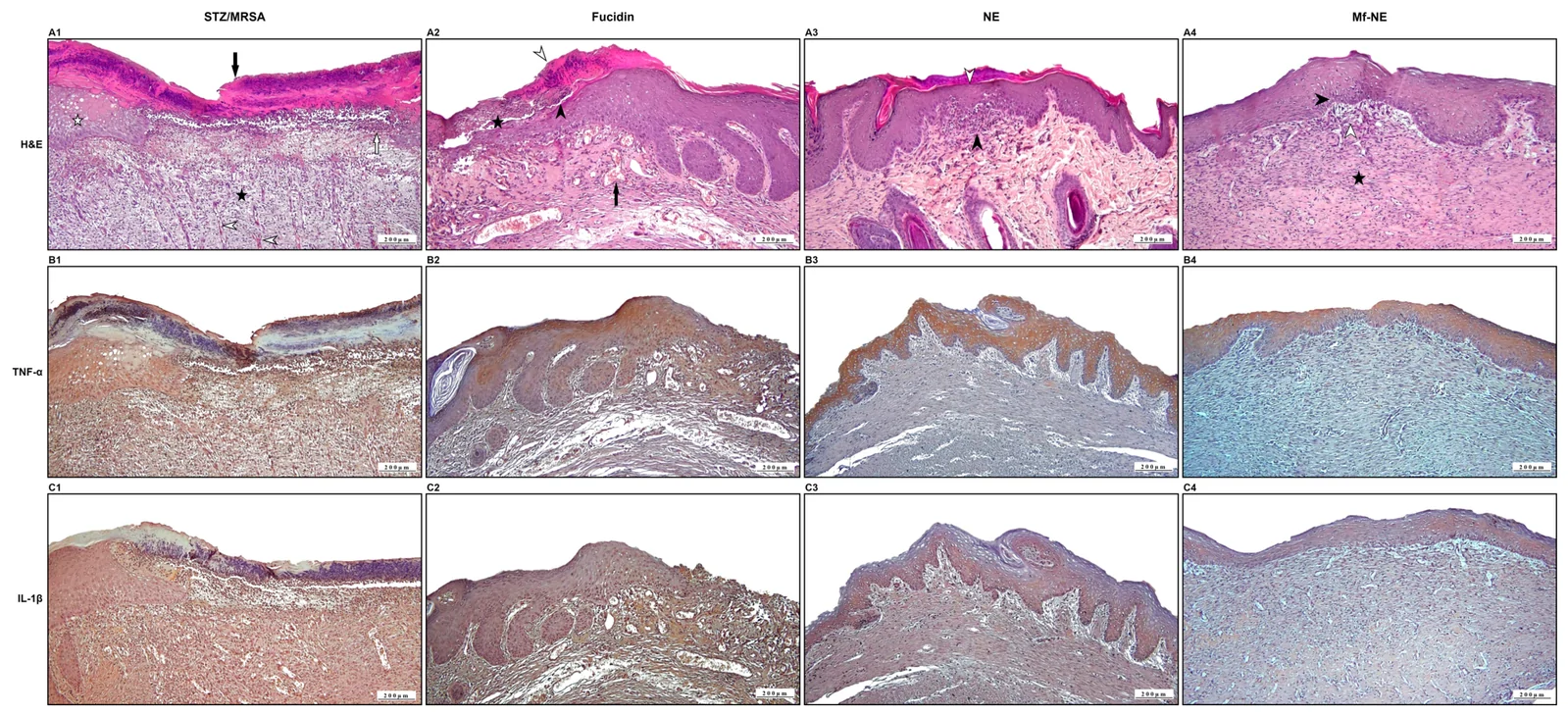
Rat skin: histopathological and immunohistochemical findings. (**A1**) STZ/MRSA group: necrotic crust in a wide area of ulceration (arrow), severe degenerative–necrotic changes and acantholysis in the epidermis (hollow star), dense inflammatory cell infiltration in the dermis and epidermis (hollow arrow), dilatation and hyperemia of the vessels (hollow arrowheads), loose collagenization (star). (**A2**) Fucidin group: limited area of ulceration and necrotic crust (hollow arrowhead), degenerative–necrotic lesions in the epidermis (star), inflammatory cell infiltration in the epidermis (arrowhead), dilatation and hyperemia of the vessels (arrow). (**A3**) NE group: mild degenerative changes in epidermal tissue integrity (hollow arrowhead) and leukocyte infiltration (arrowhead). (**A4**) Mf-NE group: very mild degenerative changes in epidermal tissue integrity (arrowhead), scant leukocyte infiltration (hollow arrowhead), dense collagenization in the dermis (star). H&E, ×100, bar: 200 µm. (**B1**) STZ/MRSA group, severe TNF-α expression. (**B2**) Fucidin group, TNF-α expression. (**B3**) NE group, TNF-α expression. (**B4**) Mf-NE group, mild TNF-α expression. (**C1**) STZ/MRSA group, severe IL-1β expression. (**C2**) Fucidin group, IL-1β expression. (**C3**) NE group, IL-1β expression. (**C4**) Mf-NE group, mild IL-1β expression. IHC, ×100, bar: 200 µm. This figure is a composite assembled from twelve separate photomicrographs, one for each group and staining combination. The panels were juxtaposed without cropping and without any adjustment of brightness, contrast, color or intensity.

In the untreated STZ/MRSA group, histopathological findings consistent with ulcerative–suppurative dermatitis were observed overall. Tissue loss due to extensive ulceration associated with severe degenerative and necrotic changes was noted in the epidermis. A diffusely distributed, severe and predominantly mononuclear leukocyte infiltration extending as far as the dermis, being more pronounced in the epidermis, was seen. Interstitial edema accompanied by poor collagenization was observed in the dermis. In addition, angiogenesis, vasodilation and hyperemia related to vascular disturbance, together with focal hemorrhage, were encountered in the dermis (Figure 9(A1)).

In the Fucidin group, histopathological findings similar to those of the untreated STZ/MRSA group were observed, but of milder severity. A more limited necrotic area in the epidermis and degenerative–necrotic lesions accompanied by mild regenerative changes were noted. Inflammatory cell infiltration was less abundant and was confined to the area of inflammation rather than being diffuse. With respect to vascular disturbance in the dermis, a decrease in angiogenesis was observed, while vasodilation and congestion were evident and hemorrhage was negligible. Collagenization in the dermis showed a tighter organization than in the untreated STZ/MRSA group, and mild granulation tissue formation was notable (Figure 9(A2)).

In the NE group, histopathological findings were determined to be milder than in the Fucidin group and particularly than in the untreated STZ/MRSA group. Despite mild degenerative changes in the epidermis, epidermal integrity was largely preserved. Inflammatory cell infiltration was scant and remained confined to the epidermal–dermal junction. Vascular changes were scarcely encountered; disorganization of the collagen fibers in the dermis was reduced and an increase in granulation connective tissue became evident (Figure 9(A3)).

In the Mf-NE group, histopathological findings were reduced to the greatest extent compared with the other groups, and an appearance close to normal skin histology was observed. Despite very mild degeneration in the epidermis, marked re-epithelialization was determined. Very slight leukocyte infiltration in the epidermis and partly in the dermis, together with mild serofibrinous exudation, was seen. While no vascular disturbance was encountered in the dermis, tightly organized collagenization and granulation tissue development were recorded (Figure 9(A4)).

Impaired healing in diabetic wounds is characterized by a prolonged inflammatory phase and delayed re-epithelialization, and this pattern has been reproduced in streptozotocin-induced diabetic rats [96]. The histopathological picture of the untreated STZ/MRSA group in the present study was consistent with this description: extensive ulceration, severe leukocyte infiltration extending into the dermis and poor collagenization were the dominant features. In contrast, marked re-epithelialization, tightly organized collagenization and the development of granulation tissue were observed in the Mf-NE group. These tissue-level findings correspond to the macroscopic wound closure reported in Section 3.7.1, and they support the suggestion made there that the formulation promotes re-formation of the epithelial tissue. Comparable histological improvement has previously been reported for *M. fruticosa* extract in an experimental burn model [17] and for an essential oil-loaded nanoemulsion in a burn wound model [20], which indicates that the histological benefit observed here is in line with earlier reports on this plant and on nanoemulsion-based topical delivery.

#### 3.7.4. Immunohistochemical Findings

On immunohistochemical examination of the expression of TNF-α, one of the major proinflammatory cytokines that plays an active role in the inflammatory phase of wound healing, severe TNF-α immunoreactivity was observed in the dermal and epidermal structures and in the inflammatory cells of the untreated STZ/MRSA group (Figure 9(B1)). In the Fucidin group, TNF-α immunoreactivity was determined to be lower than in the untreated STZ/MRSA group (Figure 9(B2)). In the NE group, TNF-α immunoreactivity was found to be lower than in the Fucidin group and particularly than in the untreated STZ/MRSA group (Figure 9(B3)). In the Mf-NE group, TNF-α immunoreactivity was observed to be markedly decreased compared with the other groups (Figure 9(B4)).

On immunohistochemical examination of the expression of IL-1β, an important stimulus for the release of proinflammatory mediators and for leukocyte migration during the initiation and conduct of the inflammatory response in wound healing, severe IL-1β immunoreactivity was seen in the dermal and epidermal structures and particularly in the inflammatory cells of the untreated STZ/MRSA group (Figure 9(C1)). In the Fucidin group, IL-1β immunoreactivity was determined to be lower than in the untreated STZ/MRSA group (Figure 9(C2)). In the NE group, IL-1β immunoreactivity was found to be lower than in the Fucidin group and particularly than in the untreated STZ/MRSA group (Figure 9(C3)). In the Mf-NE group, IL-1β immunoreactivity was recorded to be markedly decreased compared with the other groups (Figure 9(C4)).

The scoring of the histopathological and immunohistochemical findings is presented in Table 6.

**Figure 10:**
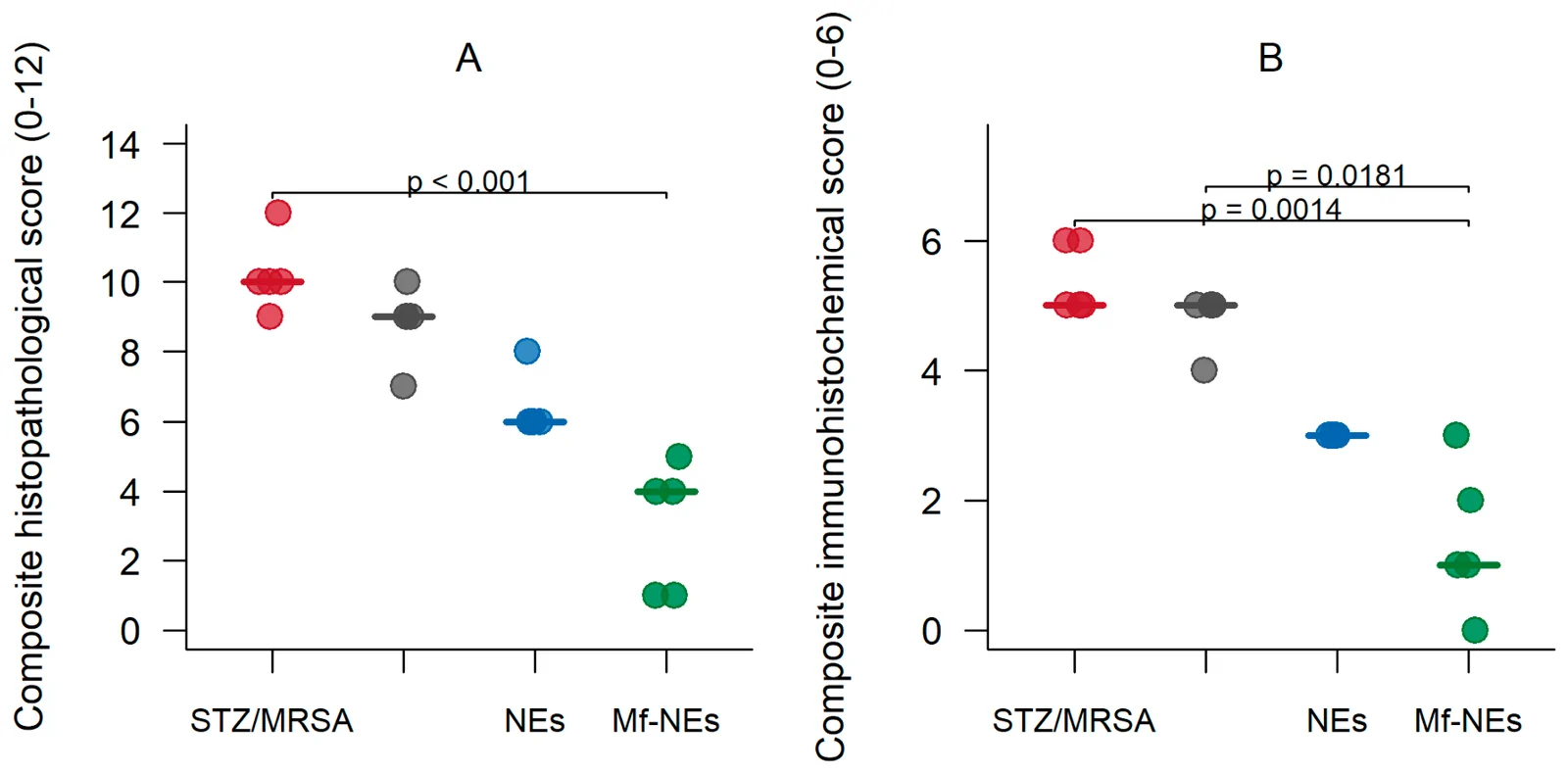
Composite histopathological and immunohistochemical scores. (**A**) Composite histopathological score (sum of erosive–ulcerative lesions, degenerative–necrotic changes, inflammatory cell infiltration and vascular disturbance grades; range 0–12). (**B**) Composite immunohistochemical score (sum of TNF-α and IL-1β grades; range 0–6). Points show individual animals (*n* = 5 per group); horizontal lines show group medians. *p* values are from Dunn’s post hoc test with Bonferroni correction. Gray points represent the Fucidin group.

The composite histopathological and immunohistochemical scores are shown in Figure 10. The composite histopathological score differed among the four groups (Kruskal–Wallis H = 16.99, *p* = 0.0007, ε^2^ = 0.89). Scores were lower in the Mf-NE group than in the untreated STZ/MRSA group (median 4, range 1–5 versus median 10, range 9–12; *p* = 0.0007), and the values of the two groups did not overlap (Cliff’s δ = 1.00). The composite immunohistochemical score followed the same pattern (H = 17.11, *p* = 0.0007, ε^2^ = 0.90): scores in the Mf-NE group (median 1, range 0–3) were lower than in the untreated STZ/MRSA group (median 5, range 5–6; *p* = 0.0014) and lower than in the Fucidin group (median 5, range 4–5; *p* = 0.0181). Neither the Fucidin group nor the NE group differed from the untreated STZ/MRSA group for either composite score. The difference between the Mf-NE and Fucidin groups in the composite histopathological score reached significance (*p* = 0.0248) but did not persist when single animals were excluded, and is therefore not reported as a difference.

TNF-α and IL-1β are among the principal mediators of the inflammatory phase of wound repair, and their sustained expression is a recognized feature of the diabetic wound environment. In both murine and human diabetic wounds, persistent IL-1β expression has been shown to prolong inflammation and to delay healing [97], while suppression of proinflammatory cytokine secretion accelerated closure in streptozotocin-induced diabetic rats [96]. In the present study, severe TNF-α and IL-1β immunoreactivity was observed in the untreated STZ/MRSA group, whereas immunoreactivity for both cytokines appeared lower in all treated groups and was least evident in the Mf-NE group. A similar association has been described in diabetic mice, where a peptide that reduced TNF-α expression was accompanied by faster wound closure and increased collagen deposition [98]. Taken together with the histopathological findings, these observations suggest that the Mf-NE formulation is accompanied by a less pronounced proinflammatory profile at the wound site, which may contribute to the transition from the inflammatory to the proliferative phase of repair. The small sample size (*n* = 5 per group) limits precision in comparisons between treatment groups. For example, all untreated STZ/MRSA animals had higher composite histopathological scores than all NE-treated animals, yet the Bonferroni-adjusted comparison was not significant (*p* = 0.0894). Non-significance does not demonstrate equivalence or absence of a treatment effect. Fucidin did not differ significantly from the untreated control in either composite score, and the individual histopathological and immunohistochemical parameters were descriptively similar between these two groups. This finding cannot be attributed solely to sample size. Strain-specific fusidic acid susceptibility results are not reported, and the description of the Fucidin regimen does not fully specify concentration, amount and frequency; these limitations prevent assessment of susceptibility-related or exposure-related explanations. Methicillin resistance alone does not establish fusidic acid resistance, and no such resistance is inferred here. Neither the descriptive appearances nor these non-significant comparisons demonstrate that the blank carrier outperforms Fucidin. In the macroscopic assessment, Fucidin had a numerically smaller remaining wound area than NE on day 12 (21.0 ± 1.9% versus 26.1 ± 1.9%); this is a distinct outcome from the histological scores.

#### 3.7.5. Gene Expression Analysis

**Figure 11:**
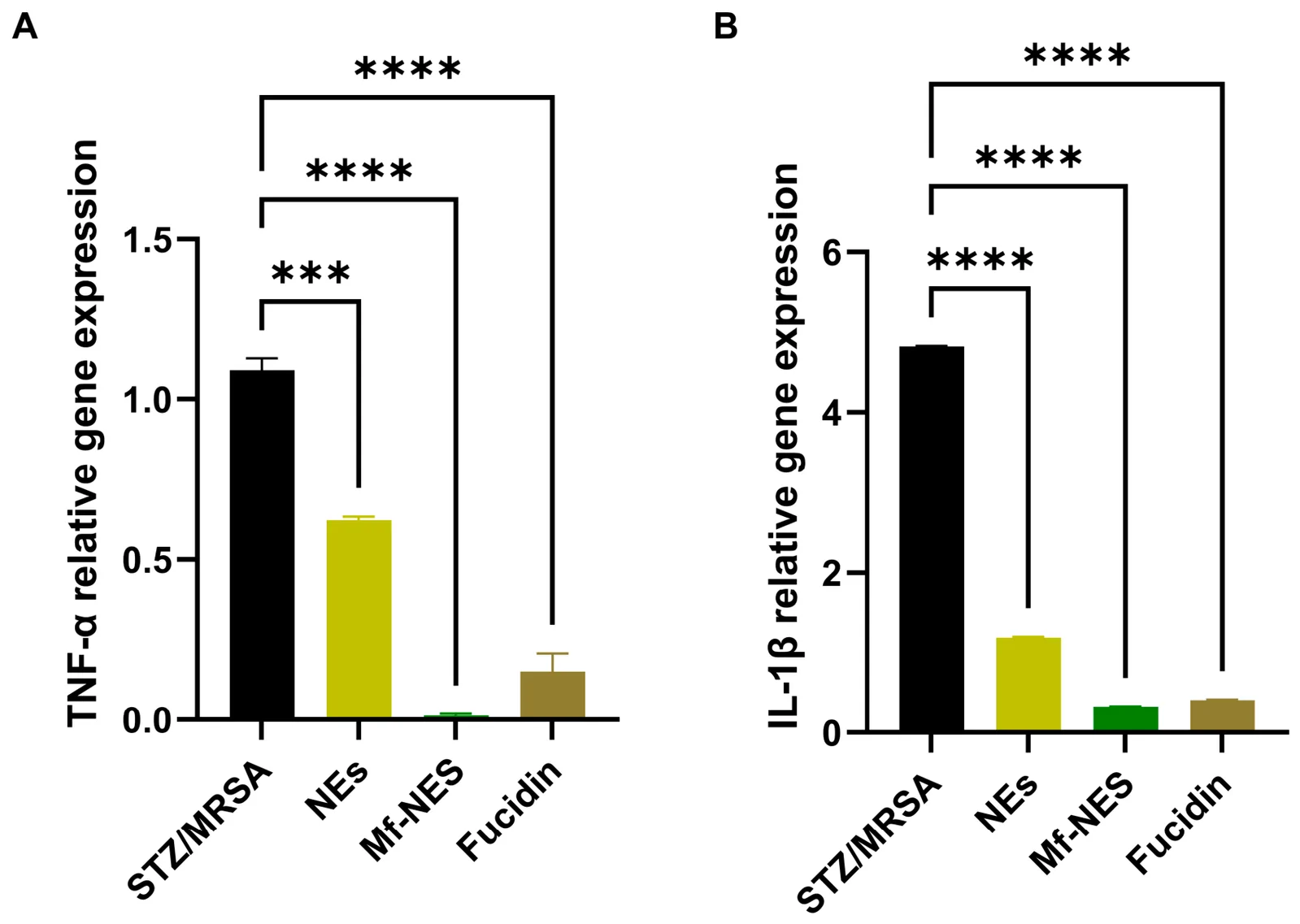
Relative gene expression of TNF-α and IL-1β in wound tissue on day 12, determined by RT-qPCR and normalized to β-actin. (**A**) TNF-α relative gene expression. (**B**) IL-1β relative gene expression. *** *p* < 0.001, **** *p* < 0.0001.

Relative gene expression of TNF-α and IL-1β in wound tissue, determined by RT-qPCR and normalized to β-actin, is shown in Figure 11. For both genes, expression was highest in the untreated STZ/MRSA group and was significantly lower in all three treated groups (NE, Mf-NE and Fucidin), with the lowest relative expression observed in the Mf-NE group (TNF-α: STZ/MRSA vs. NE, *p* < 0.001; STZ/MRSA vs. Mf-NE and vs. Fucidin, *p* < 0.0001; IL-1β: STZ/MRSA vs. NE, Mf-NE and Fucidin, *p* < 0.0001 for each).

The decrease in TNF-α expression in the NE group relative to the untreated STZ/MRSA group suggests that application of the blank nanoemulsion (NE) may partially suppress the inflammatory response. In contrast, the far more pronounced reduction in TNF-α expression in the Mf-NE and Fucidin groups suggests that the chronic proinflammatory stimulus was largely abolished in the treated wounds. A similar relationship between a decrease in TNF-α and accelerated wound closure has been reported. For example, Suliman and colleagues showed in 2025 that treatment with an SDF-1α-containing collagen-based hydrogel accelerated wound closure in diabetic rats while significantly reducing TNF-α and IL-1β gene expression [99]. Similarly, Rajić and colleagues reported that inflammatory cell infiltration and the mRNA expression of proinflammatory genes were decreased in diabetic rat wounds treated with plasma-activated water, and that these changes were associated with faster healing [36,99]. In a 2023 study, Zhang et al. showed that in diabetic rats treated with paeonol the decrease in TNF-α and IL-1β levels was accompanied by reduced inflammatory cell infiltration and by increased collagen deposition and VEGF/CD31-associated angiogenesis [100]. Accordingly, the marked suppression of TNF-α together with the wound closure seen in the Mf-NE group in our study suggests that a more effective transition from the inflammatory to the proliferative phase may have taken place. Similarly, ref. [101] evaluated TNF-α expression by qRT-PCR in the wound tissue of STZ-induced diabetic rats and reported that, on day 21, the TNF-α level in the untreated diabetic control group (12.30 ± 5.93) was numerically higher than in the treated diabetic groups. This finding parallels the marked decrease in TNF-α expression observed after Mf-NE application in our study and supports the possibility that effective wound healing is linked to suppression of the chronic proinflammatory response.

In a 2024 study by Anuradha and colleagues using a ferulic acid-containing nanoemulgel in diabetic rats, the nanoformulation was shown to reduce inflammatory markers including TNF-α and IL-1β, accompanied by increased granulation tissue formation and wound healing [102]. Current studies in diabetic rats, as seen in the 2025 study by Suliman and colleagues, show that a decrease in IL-1β occurs together with a reduced inflammatory cell burden, increased collagen formation and accelerated wound contraction. In that study, the treatment that produced the strongest wound healing was also the treatment that lowered TNF-α and IL-1β gene expression the most, which is strikingly similar to the findings for the Mf-NE group in our study [99].

## 4. Conclusions

In the present study, an essential oil-loaded nanoemulsion was developed from *M. fruticosa* and evaluated as a topical treatment for MRSA-infected diabetic foot wounds. The essential oil was rich in oxygenated monoterpenes, and its incorporation into the nanoemulsion yielded a low-viscosity formulation with Newtonian flow behavior and a skin-compatible pH. Loading the oil conferred antibacterial activity against MRSA that the blank carrier did not possess, and it substantially increased radical scavenging capacity. The Mf-NE formulation also demonstrated good biocompatibility in L929 fibroblast cells. In the animal model, the Mf-NE group showed the smallest residual wound area at day 12, near-complete suppression of bacterial colonization of the wound, and the histological appearance closest to normal skin. TNF-α and IL-1β gene expression levels were significantly decreased in the wound tissue. Semi-quantitative scoring placed these observations on a measured footing: both the composite histopathological score and the composite score for TNF-α and IL-1β immunoreactivity were lower in the Mf-NE group than in the untreated group, with no overlap between the two groups. Taken together, these results indicate that nanoemulsification is an effective strategy for the topical delivery of *M. fruticosa* essential oil to infected diabetic wounds, bringing antibacterial, antioxidant and inflammation-related effects together in a single formulation. The principal limitation of the study is the group size of five animals, which leaves it underpowered for comparisons between adjacent treatment groups. The formulation was not characterized morphologically by transmission electron microscopy, and long-term storage stability was not assessed; both are acknowledged as limitations and are recommended for future work. Stability and dermal safety studies will also be required before clinical translation. The present results do not establish a safe topical pulegone exposure, and the Fucidin control requires fuller dosing and susceptibility information for interpretation.

## Figures and Tables

**Table 1:** Primer sequences used for RT-qPCR analysis of TNF-α, IL-1β and β-actin.

Oligonucleotide ID	Nucleotide Sequence	Source
IL-1β	F: 5′-AGCAGCTTTCGACAGTGAGG-3′	[36]
	R: 5′-CTCCACGGGCAAGACATAGG-3′	
TNF-α	F: 5′-GCCACCACGCTCTTCTGTCT-3′	
	R: 5′-CGCTTGGTGGTTTGCTACGAC-3′	
β-actin	F: 5′-CTCTGTGTGGATCGGTGGCT-3′	[37]
	R: 5′-GCAGCTCAGTAACAGTCCGC-3′	

**Table 2:** Chemical composition of *M. fruticosa* essential oil determined by GC–MS analysis.

Monoterpene Hydrocarbons			
Compound	RT	RI (DB-Wax)	% Composition
β-Pinene	14.748	1122	0.93
D-Limonene	19.267	1228	1.05
**Oxygenated Monoterpenes**			
**Compound**	**RT**	**RI (DB-Wax)**	**% Composition**
Isomenthone	32.277	1527	9.4
Pulegone	38.954	1683	42.81
Piperitone	41.861	1763	5.38
*p*-Mentha-1,8-dien-3-one (+)	45.338	1870	0.44
Piperitenone	47.816	1958	29.73
**Sesquiterpene Hydrocarbons**			
**Compound**	**RT**	**RI (DB-Wax)**	**% Composition**
Bicyclogermacrene	42.643	1785	1.59
**Other Compounds**			
**Compound**	**RT**	**RI (DB-Wax)**	**% Composition**
1-Octanol	34.782	1585	1.12
Hexadecane	57.294	2369	4.75
1-Hexadecanol, 2-methyl-	60.465	2510	1.77
Hexaethylene glycol monododecyl ether	61.788	2562	1.04

**Table 3:** Composition of optimum nanoemulsion formulation (S/CoS ratio 2:1).

Components (%)/Formulations	Blank NE (NE)	Mf-NE (5%)
*Micromeria* essential oil	-	5
Oleic Acid	15.331	10.331
Cremophor^®^ EL	19.360	19.360
Tween 80	19.360	19.360
Transcutol^®^	19.360	19.360
Distilled Water	26.588	26.588

NE represents the blank nanoemulsion without *M. fruticosa* essential oil, whereas Mf-NE contains 5% (*w*/*w*) *M. fruticosa* essential oil, replacing an equivalent portion of the oleic acid phase.

**Table 4:** The physical properties of the nanoemulsion formulations (mean ± SD, *n* = 3).

Formulation	Droplet Size (nm ± SD)	PDI ± SD	Zeta Potential (mV ± SD)	Conductivity (mS/cm ± SD)
Blank NE (NE)	214.5 ± 14.4	0.402 ± 0.01	0.0 ± 0.1	0.204 ± 0.02
Mf-NE (5%)	292.9 ± 9.9	0.560 ± 0.01	0.0 ± 0.1	0.201 ± 0.01

**Table 5:** Viscosity and pH values of nanoemulsion at 25 °C (mean ± SD, *n* = 3).

Formulation	pH ± SD	Viscosity (mPa·s ± SD)
Blank NE (NE)	5.44 ± 0.1	42.33 ± 2.7
Mf-NE (5%)	5.92 ± 0.1	29.49 ± 1.6

**Table 6:** Semi-quantitative scoring of histopathological and immunohistochemical findings (*n* = 5 per group).

Group	Animal No.	EUL	DNC	ICI	VD	TNF-α	IL-1β
STZ/MRSA	1	+++	+++	+++	+++	+++	+++
STZ/MRSA	2	+++	+++	++	++	+++	++
STZ/MRSA	3	++	+++	++	+++	++	+++
STZ/MRSA	4	+++	++	+++	++	+++	+++
STZ/MRSA	5	++	+++	++	++	+++	++
Fucidin	1	++	+++	++	++	+++	++
Fucidin	2	+++	++	+	+++	++	+++
Fucidin	3	+++	++	+++	++	+++	++
Fucidin	4	+	+++	++	+	++	++
Fucidin	5	++	++	+++	++	++	+++
NE	1	++	+	++	+	++	+
NE	2	+	++	++	+	++	+
NE	3	++	+	+	++	+	++
NE	4	++	+++	+	++	++	+
NE	5	+	++	++	+	+	++
Mf-NE	1	−	−	+	−	+	−
Mf-NE	2	+	−	++	+	−	+
Mf-NE	3	−	+	−	−	−	−
Mf-NE	4	+	++	+	+	+	++
Mf-NE	5	++	+	+	−	+	+

EUL, erosive–ulcerative lesions; DNC, degenerative–necrotic changes; ICI, inflammatory cell infiltration; VD, vascular disturbance. Grading: −, absent; +, mild; ++, moderate; +++, severe.

## Data Availability

The data generated and/or analyzed during the current study are available from the corresponding author upon reasonable request.

## Abbreviations

ABTS, 2,2′-azino-bis(3-ethylbenzothiazoline-6-sulfonic acid); AMR, antimicrobial resistance; ATCC, American Type Culture Collection; CFU, colony-forming unit(s); DFU, diabetic foot ulcer; DLS, dynamic light scattering; DPPH, 2,2-diphenyl-1-picrylhydrazyl; EO(s), essential oil(s); GC–MS, gas chromatography–mass spectrometry; IL-1β, interleukin-1 beta; MHA, Mueller–Hinton agar; Mf-NE(s), *Micromeria fruticosa* essential oil-loaded nanoemulsion(s); MIC, minimum inhibitory concentration; MRSA, methicillin-resistant *Staphylococcus aureus*; NE(s), blank (essential oil-free) nanoemulsion(s); NIST14, National Institute of Standards and Technology mass spectral library (2014 edition); PBS, phosphate-buffered saline; PDI, polydispersity index; ROS, reactive oxygen species; SD, standard deviation; SPF, specific pathogen-free; STZ, streptozotocin; TNF-α, tumor necrosis factor alpha.
